# Antioxidants by nature: an ancient feature at the heart of flavonoids' multifunctionality

**DOI:** 10.1111/nph.20195

**Published:** 2024-10-21

**Authors:** Giovanni Agati, Cecilia Brunetti, Luana Beatriz dos Santos Nascimento, Antonella Gori, Ermes Lo Piccolo, Massimiliano Tattini

**Affiliations:** ^1^ Institute of Applied Physics ‘Carrara’ (IFAC) National Research Council of Italy Via Madonna del Piano 10 I‐50019 Sesto Fiorentino, Florence Italy; ^2^ Institute for Sustainable Plant Protection (IPSP) National Research Council of Italy Via Madonna del Piano 10 I‐50019 Sesto Fiorentino, Florence Italy; ^3^ Federal University of Rio de Janeiro (UFRJ) Ave Carlos Chagas Filho, s/n–CCS Rio de Janeiro 21941‐590 Rio de Janeiro Brazil; ^4^ Department of Agri‐Food Production and Environmental Sciences (DAGRI) University of Florence Viale delle Idee 30 I‐50019 Sesto Fiorentino, Florence Italy

**Keywords:** abiotic and biotic stresses, angiosperms, auxin, bryophytes, flavonoids, multifunctionality, oxidative stress signaling, UV radiation

## Introduction

Early land plants' ability to adapt to novel environmental pressures associated with an ever‐changing terrestrial habitat was the result of a vast set of evolutionary innovations, including metabolic ones (Wagner, 2011; Bowman *et al*., 2017). Land plants, as sessile organisms, were driven to evolve integrated and modular metabolic pathways. Several of them were true metabolic network innovations, responsible for synthesizing several novel compounds (Cannell *et al*., 2020; Dadras *et al*., 2023b). The new specialized metabolites (SMs) contributed to thrive in these new and frequently hostile environments (Rensing, 2018; Cheng *et al*., 2019; Han *et al*., 2019; Buschmann, 2020; Fürst‐Jansen *et al*., 2020). There is evidence that metabolic plasticity is a key component of a highly complex network in the plant–environment interaction, which also includes morphoanatomical traits. This network largely and ultimately determines the ability of terrestrial plants to escape from the most severe environmental threats, the so‐called ‘flight strategy’ of sessile organisms (Potters *et al*., 2007; Lauder *et al*., 2019). While an elaborate metabolic system was already placed in the closest algal ancestors of land plants (Rieseberg *et al*., 2021; Dadras *et al*., 2023a), primary and particularly secondary metabolic networks have grown far more sophisticated throughout plant evolution (Keeling *et al*., 2010; Wang *et al*., 2015; Maeda, 2019; Bowles *et al*., 2020; Li *et al*., 2024). They contributed to land plant distribution toward more challenging habitats (Steemans *et al*., 2009). For instance, the R2R3MYB family of transcription factors (TFs), which regulates a wide array of biological processes, including the expression of genes involved in the biosynthesis of phenylpropanoids, has been extraordinarily expanded and diversified in the lineage of angiosperms (Feller *et al*., 2011; Bowman *et al*., 2017; Albert *et al*., 2018; Jiang & Rao, 2020; Davies *et al*., 2021). Enzymes involved in both the ‘decoration’ of basic phenylpropanoid skeletons (e.g. the C6‐C3‐C6 core skeleton of flavonoids) and their transport to different subcellular compartments have also expanded much throughout plant evolution (Kitamura, 2006; Tohge *et al*., 2018; Alseekh *et al*., 2020; Davies *et al*., 2020; Li *et al*., 2020; Wen *et al*., 2020). The extraordinary chemical diversity originated from the rise and evolution of multiple SM pathways, coupled with their location in different tissues and cellular compartments, well explains the outstanding plant adaptability to harsh stressful conditions (*sensu stricto*, that is, distance from pre‐existing homeostasis) associated with the terrestrial habitat (Fürst‐Jansen *et al*., 2020; Rensing, 2020).

The pivotal role of SMs in the adaptability of land plants depends not only on their extraordinarily high number and diversified skeletons, synthesized by different taxa (Weng *et al*., 2021), but also on their inherent ability to play multiple functions (Milo & Last, 2012; Ehlers *et al*., 2020; Mutwil, 2020; Durán‐Medina *et al*., 2021; Hu *et al*., 2021; de Vries *et al*., 2021; Weng *et al*., 2021). Although SM biosynthesis might have served as a sink for the excess of carbon available to plants during their initial exploration of a highly enriched CO_2_ atmosphere (Dadras *et al*., 2023a,b), SMs multifunctionality efficiently compensates for the energetic cost required for their biosynthesis (Kliebenstein, 2013; Erb & Kliebenstein, 2020). The multifunctional nature of SMs and their high responsiveness to abiotic and biotic stressors provide plants with an unlimited defense arsenal, in which each SM may play different roles depending on the severity of the stress events and the degree of plant body complexity. These factors determine the metabolite distribution at the organ, tissue, cellular, and subcellular levels (Schneider *et al*., 2019; Wang *et al*., 2019; Shitan & Yazaki, 2020; Weng *et al*., 2021). In simpler terms, the evolution of multifunctional SM biosynthesis follows the natural tendency to catch as many flies with one clamp as possible (Wink, 1999; Izhaki, 2002).

**Fig. 1:**
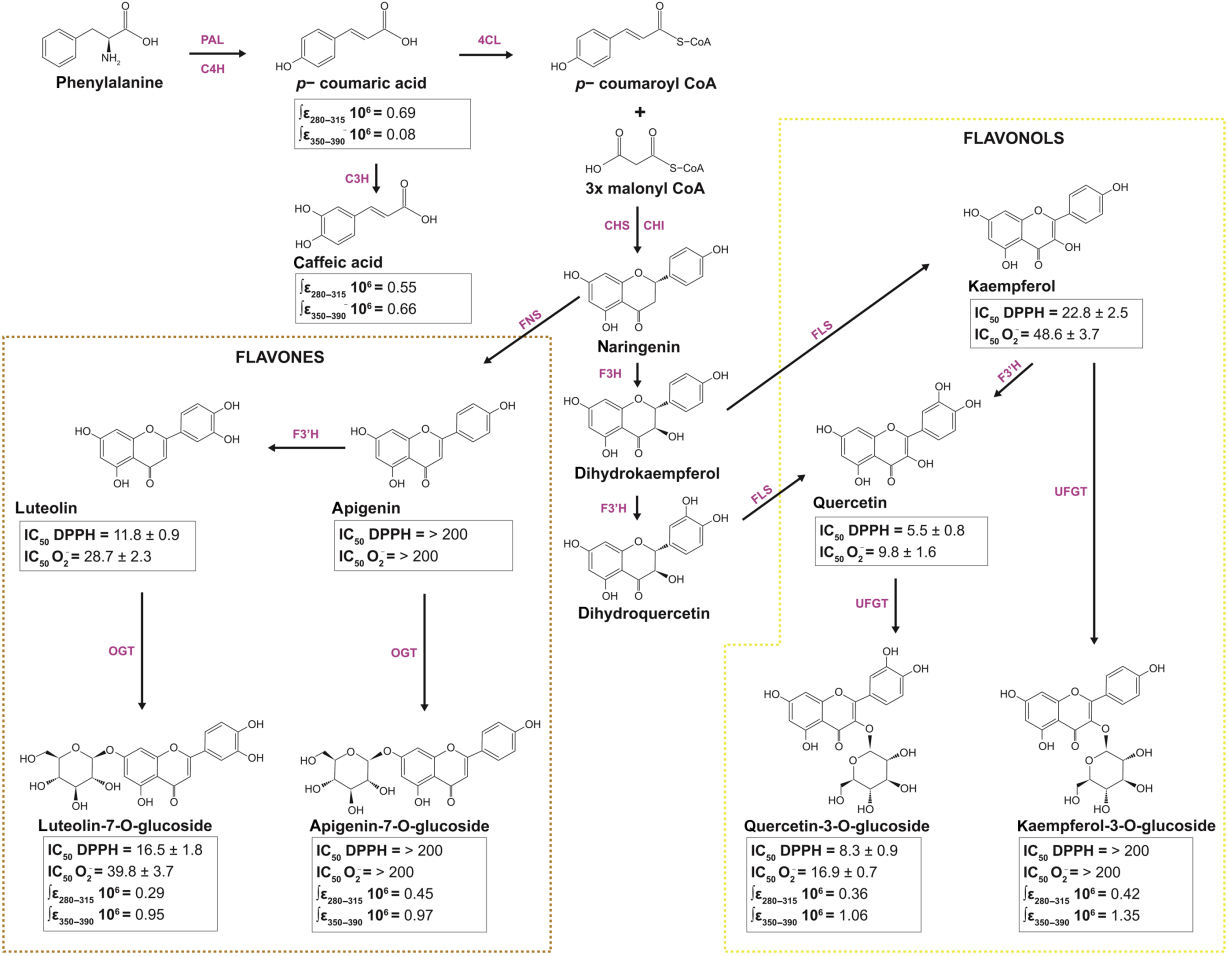
Simplified scheme of the phenylpropanoid pathway leading to the biosynthesis of hydroxycinnamic acid derivatives (HCAs, here reported are *p*‐coumaric and caffeic acids), mono‐ and dihydroxy B‐ring‐substituted flavones and flavonols (FLAV). The UV‐absorbing capacity of HCAs and FLAV has been measured by integrating individual molar extinction coefficients (ε) over the 280–315 (UV‐B) and 315–390 nm (UV‐A) waveband. The antioxidant capacity of FLAV, both aglycones and glycoside derivatives, has been estimated by calculating the concentration (μM) of individual metabolites capable of reducing by 50% (IC_50_) that of the synthetic free radical DPPH (2,2‐diphenyl‐1‐picrylhydrazyl) and the superoxide anion (O_2_^−^), following the spectrophotometric protocols of Baratto *et al*. (2003). Data of IC_50_ are means ± SD of three replicate measurements. 4CL, 4‐coumaroyl‐CoA ligase; C3H, *p*‐coumarate 3‐hydroxylase; C4H, cinnamate 4‐hydroxylase; CHI, chalcone isomerase; CHS, chalcone synthase; F3′H, flavonoid 3′‐hydroxylase; F3H, flavanone 3‐hydroxylase; FLS, flavonol synthase; FNS, flavone synthase; OGT; 7‐*O*‐glucosyl transferase; PAL, phenylalanine ammonia‐lyase; UFGT; UDP glucose‐flavonoid 3‐*O*‐glucosyl transferase.

Here, we focus on the ancient and ubiquitous class of flavonoids (Fig. 1), which are highly responsive to abiotic and biotic environmental stressors and are capable of regulating key steps in plant growth and development (Pollastri & Tattini, 2011; Schneider *et al*., 2019; Chapman & Muday, 2021; Garagounis *et al*., 2021; Venegas‐Molina *et al*., 2021; Daryanavard *et al*., 2023). However, their multifunctionality makes it difficult to determine the foremost environmental drivers for the emergence and diversification of the flavonoid metabolic network, despite decades of extensive research (Rozema *et al*., 1997, 2002; Buer *et al*., 2010; Tripp *et al*., 2018; Yonekura‐Sakakibara *et al*., 2019; Davies *et al*., 2020). We provide a detailed analysis of the complex relationship between the multifunctional nature of flavonoids and the environmental stimuli primarily responsible for the rise of the flavonoid metabolic network, offering conclusive evidence for the structural–functional relationship that is at the root of their functional versatility.

## Did flavonoid metabolism first emerge in response to biotic pressures?

The emergence of flavonoids represented an outstanding major metabolic innovation during the plants' water‐to‐land transition (de Vries *et al*., 2017; Davies *et al*., 2020; Dos Santos Nascimento & Tattini, 2022). This rise has been initially hypothesized to have occurred in response to herbivore pressure (Swain, 1977; Cooper Driver, 1980), the long‐known ‘biochemical coevolutionary arms–race theory’ (Ehrlich & Raven, 1964). In brief, the rise and the diversification of flavonoids, in terms of number and structural complexity, paralleled with major changes in plant morphology, would have been a direct consequence of the selective pressure caused by predation and diseases (Levin, 1971; Swain, 1975, 1977). This coevolution hypothesis has been proven for several classes of SMs, but questioned in other instances, such as the case of flavonoids and other phenolics (Jones & Firn, 1991; Close & McArthur, 2002; Davies *et al*., 2020; Erb & Kliebenstein, 2020). For instance, Rausher (2001) argued that plant enemies are too rare to generate a frequent evolution of defensive features, such as the biosynthesis of many SMs, particularly flavonoids. Close & McArthur (2002) pointed out the relatively minor role of many phenolics, including flavonoids, as anti‐herbivore agents, while providing evidence for their main functions as photo‐protectants. Although tannins have historically been viewed as defense compounds against herbivore insects, relatively new evidence supports their antioxidant role (Salminen & Karonen, 2011; Constabel *et al*., 2014; Gourlay & Constabel, 2019). Finally, the vast literature concerning the phenylpropanoid biosynthesis in response to herbivores and their role in plant resistance has not provided proof of the predominant role of flavonoids as deterrents for herbivores (Serrano *et al*., 2012; Garcia‐Molina & Pastor, 2024). For instance, UV‐B radiation, which is known to trigger flavonoid biosynthesis, has been reported to either increase or decrease the resistance to herbivores in a range of species (Izaguiree *et al*., 2003; Rousseaux *et al*., 2004; Schneider *et al*., 2019). The biosynthesis of flavonoids is strongly suppressed by the bacterial *flg22*, which indeed stimulates other phenylpropanoid biosynthetic branch pathways (Serrano *et al*., 2012), in agreement with the observation that sinapic and caffeic acid derivatives offer higher herbivory resistance than flavonoids (for a review, see Ballaré, 2014). There is also convincing evidence that most angiosperms prioritize immune responses over stress‐induced flavonoid accumulation under microbial attack, and this might represent an ancient evolutionary regulatory crosstalk mechanism (Lozoya *et al*., 1991; Lo & Nicholson, 1998; Logemann & Hahlbrock, 2002; Serrano *et al*., 2012).

It is conceivable that, despite flavonoids' excellent antibacterial properties, resistance to natural enemies driven by greater production of these compounds may merely be a side consequence of chemicals that evolved to perform other ecological purposes (Rausher, 2001; Erb & Kliebenstein, 2020). This hypothesis is reasonable based on both the multifunctional nature of SMs and the vast range of environmental stresses, other than predators, that plants face on land (Rensing, 2018; Donoghue *et al*., 2021).

## The intriguing relationship between flavonoids and oxidative stress

It is worth noting that once plants moved onto land, they were confronted with a novel set of abiotic environmental stresses, such as the scarcity of water and nutrients, high solar irradiance and changing spectral quality of light, and huge fluctuation in air temperature (Fürst‐Jansen *et al*., 2020; Markham & Greenham, 2021; Xu *et al*., 2021; Kim *et al*., 2022). The evolution of a molecular network conferring water stress resistance is indeed the typical feature of all land plants (Rensing, 2020; Schreiber *et al*., 2022). This supports the view that the simultaneous action of abiotic stressors, predominantly but not exclusively a combination of water scarcity and high sun irradiation, was the fundamental driver for the rise of SM biosynthesis pathways, including for flavonoids (Rensing, 2018; Brunetti *et al*., 2019; Dixon & Dickinson, 2024). Flavonoid biosynthesis is greatly activated in response to drought stress and high solar irradiation (Tattini *et al*., 2004, 2015; Nakabayashi *et al*., 2015; Siipola *et al*., 2016; Wang *et al*., 2020), but it is also triggered by nutrient deficiency, salinity and cold (Lillo *et al*., 2008; Agati *et al*., 2011; Albert *et al*., 2018; Bian *et al*., 2019; Sachdev *et al*., 2021). This leads to the hypothesis that changes in reactive oxygen species (ROS)/redox homeostasis, as commonly occur in plants exposed to a wide range of environmental stressors (Devireddy *et al*., 2021; Peláez‐Vico *et al*., 2022; Dietz & Vogelsang, 2024), may have regulated the flavonoid biosynthesis in land plants (Babu *et al*., 2003, 2005; Taylor & Grotewold, 2005; Xu *et al*., 2015). This hypothesis fits well with the notion that the activities of most TFs that regulate the flavonoid biosynthetic genes, including the R2R3MYBs, are under tight ROS/redox control (Heine *et al*., 2004; He *et al*., 2018; Imran *et al*., 2018; Martin *et al*., 2022; Pratyusha & Sarada, 2022). There is compelling evidence that stress‐induced increase in the excitation pressure on PSII and the subsequent change in the redox status of the photosynthetic electron transport chain (PETC) serve as retrograde signals (chloroplast‐to‐nucleus signaling) to regulate flavonoid biosynthesis (Gerhardt *et al*., 2008; Akhtar *et al*., 2010; Richter *et al*., 2020, 2023).

While ROS/redox regulation of flavonoid production does not necessarily point to a primary function of these molecules as quenchers/scavengers of stress‐induced ROS accumulation (i.e. as antioxidants *sensu stricto*), flavonoids are components of the integrated antioxidant network, aimed at keeping the ROS level within a sub‐lethal concentration range, under the most severe stressful conditions (to be described later, for details, Agati *et al*., 2007, 2012, 2020; Nakabayashi *et al*., 2015; Tattini *et al*., 2015; Muhlemann *et al*., 2018; Chapman & Muday, 2021; Martin *et al*., 2022). We note that the addition of far‐red light (FR), which is known to induce a more oxidized PETC, inhibits the biosynthesis of flavonoids and greatly decreases the ratio of quercetin (Que) to kaempferol (Kae) derivatives, which is the inverse of what happens when *Brassica napus* is supplemented with UV‐B radiation (Gerhardt *et al*., 2008). On the contrary, several studies have observed a marked increase in Que to Kae ratio in several angiosperms, such as pea, soybean, and *Arabidopsis thaliana*, supplemented with red light (R) (Furuya *et al*., 1962; Falcone Ferreyra *et al*., 2021; Lim *et al*., 2023). An increase in Que to Kae derivatives, or in dihydroxy B‐ring (dihydroxy thereafter) to monohydroxy B‐ring‐substituted (monohydroxy) flavonoids, is commonly observed in plant lineages of different complexity (such as bryophytes and angiosperms) in response to a wide range of abiotic stressors, including to high PAR and UV‐B radiation (for review articles see, Pollastri & Tattini, 2011; Neugart & Schreiner, 2018; Agati *et al*., 2020; Davies *et al*., 2020; Dos Santos Nascimento & Tattini, 2022; Singh *et al*., 2023). While Que and Kae aglycones, the last to a considerably lesser extent, have an effective ability to scavenge free radicals and ROS, this is not the case for Kae derivatives, in which the highly reactive 3‐OH (flavonol) group is usually glycosylated (Rice‐Evans *et al*., 1996; Fig. 1). Glycosylation makes flavonoids soluble in the aqueous cellular milieu, prevents their auto‐oxidation, facilitates their transport from the endoplasmic reticulum (ER) to different cellular compartments, but depresses to some extent their antioxidant capacity (Fig. 1). The ROS‐scavenging activity of flavonoids mostly depends on the presence of the catechol group in the B‐ring, followed by the presence of both C2‐C3 unsaturation and a 4‐oxo function in the C‐ring, just like in Que (Rice‐Evans *et al*., 1996; Williams *et al*., 2004). Consistently, Que 3‐*O*‐glucoside has a lower ROS‐scavenging ability than Que, but considerably higher antioxidant capacity than Kae, whereas Kae 3‐*O*‐glucoside displays negligible antioxidant capacity (Fig. 1). While we cannot rule out the possibility that glycosylated flavonoids are de‐glycosylated, releasing the most active aglycone forms (e.g. plants contain a plethora of β‐glucosidase that may perform this function, Roepke & Bozzo, 2015; Le Roy *et al*., 2016; Baba *et al*., 2017), there is no consistent body of evidence showing the presence of flavonoid aglycones in plant cells prone to oxidative stress, such as in epidermal and sub‐epidermal tissues (Wollenweber *et al*., 2011; Ketudat Cairns *et al*., 2015; Baba *et al*., 2017; Uehara *et al*., 2018).

**Fig. 2:**
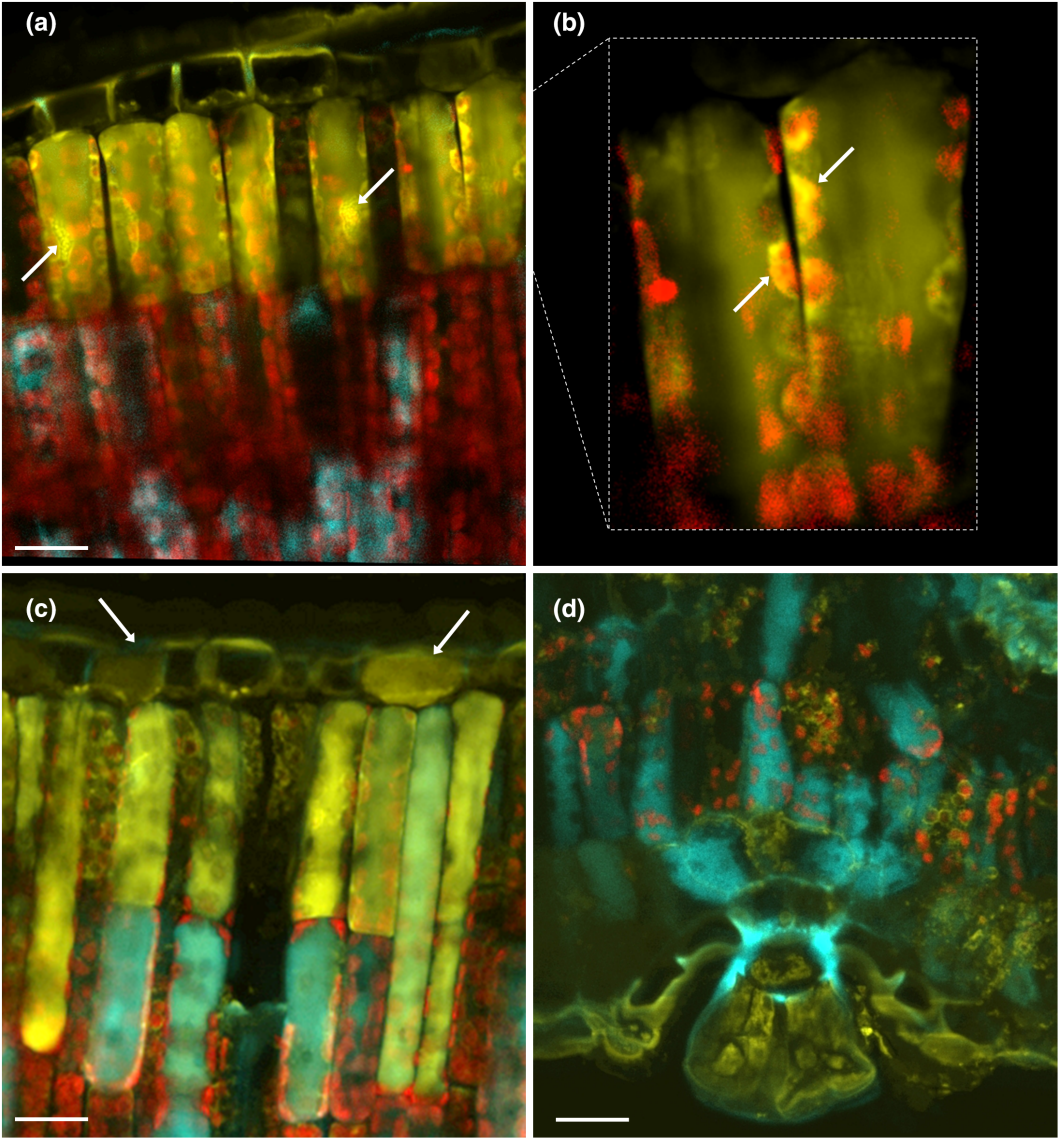
Inter‐ and intra‐cellular distribution of flavonoids (FLAV) and hydroxycinnamic acid derivatives (HCAs) in 6‐month‐old *Phillyrea latifolia* leaves newly developed in full sunlight. Cross sections were stained with Naturstoff reagent (NR, phosphate‐buffered (pH 6.8) saline (1%, w/v, NaCl) solution of 0.1% (w/v) 2‐amino ethyl diphenyl boric acid) and merged fluorescence images (a–d) result from confocal laser scanning microscopy (CLSM) analysis under the following, sequential, excitation (exc)/emission (em) setups. λ_exc_ = 365/λ_em_ = 415–485 nm for HCA‐derived blue fluorescence; λ_exc_ = 488/λ_em_ = 565–535 nm for FLAV‐derived yellow fluorescence; λ_exc_ = 638/λ_em_ = 690–785 nm for chlorophyll‐derived red fluorescence. FLAV accumulate in the vacuoles and the nuclei of adaxial parenchyma (arrows in a), in the outer envelope membranes of the chloroplasts (arrows in b), and in the vacuoles of adaxial epidermal cells (arrows in c). HCAs occur in abaxial mesophyll cells, which have a palisade‐like morpho‐anatomy (as typically occurs in sun‐adapted leaves), together with yellow fluorescent FLAV (d). The multicellular glandular trichome exclusively accumulates FLAV in the vacuole and likely in the cytoplasm, whereas HCAs are merely distributed in the wall of the trichome stalk cell (d). Bars, 20 μm.

**Fig. 3:**
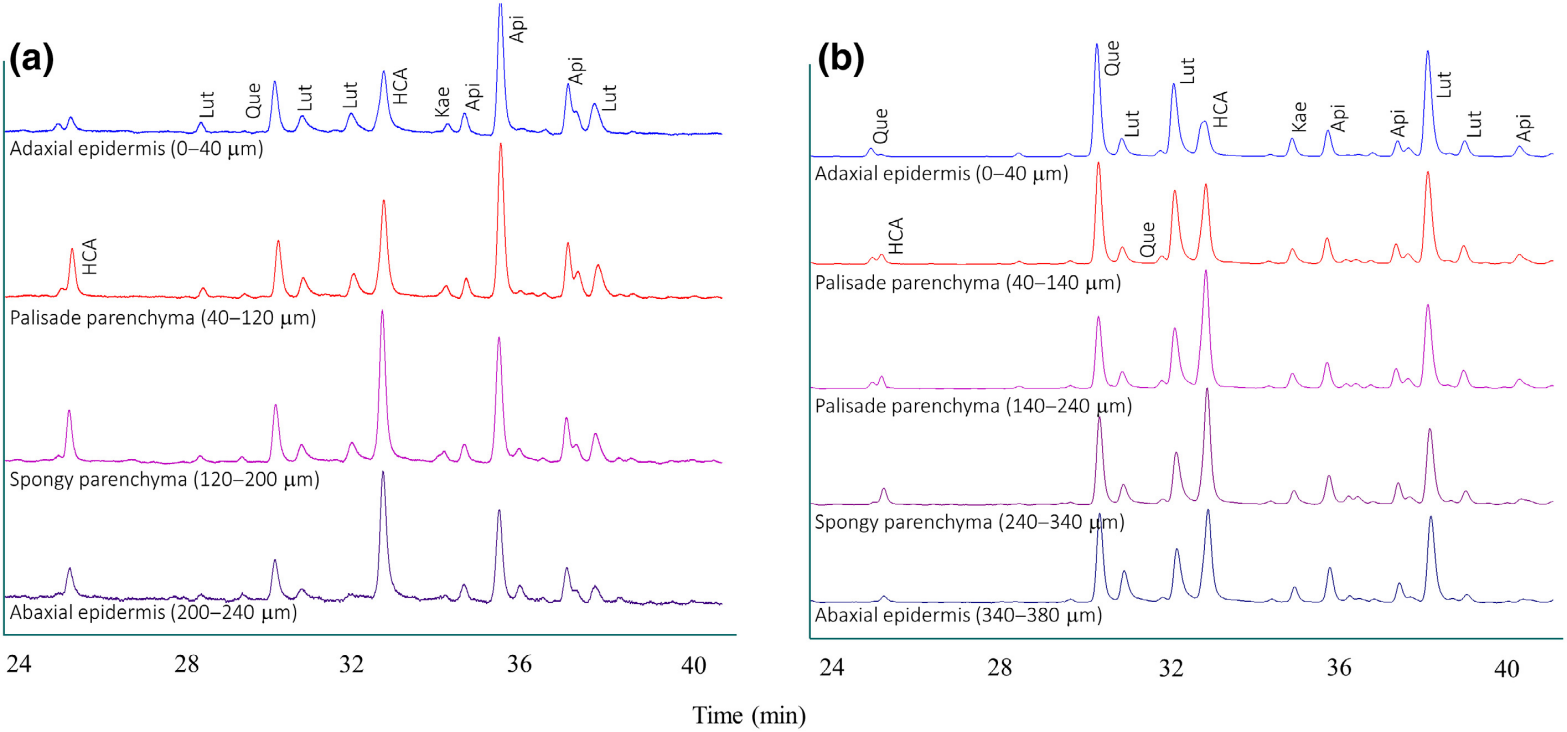
Representative high performance liquid chromatography (HPLC)‐DAD chromatograms of different tissue layers of 3‐month‐old *Phyllirea latifolia* leaves newly developed in partial shading (25% full sunlight, a) or in full sunlight (b), showing large light‐induced changes in phenylpropanoid composition. While hydroxycinnamic acid (HCA) and apigenin (Api) derivatives mostly contribute to the phenylpropanoid pool in shaded leaves, quercetin (Que) and luteolin (Lut) derivatives largely represent the phenylpropanoids synthesized by full‐sun exposed leaves. Of note, HCAs accumulate poorly in the mostly light‐exposed adaxial epidermis in leaves exposed to the greatest UV‐B irradiance (b), despite their greatest ability to absorb solar UV‐B wavelengths. Longitudinal sections were cut with a cryo‐microtome following the protocols of Ålenius *et al*. (1995) and Tattini *et al*. (2015) from leaves sampled at midday. The quali‐ and quantitative analysis of phenylpropanoids were performed using the protocol of Tattini *et al*. (2015) and chromatograms (recorded at 350 nm) were normalized based on the fresh weight of different tissue layers.

The functional significance of flavonoids as antioxidants in an *in planta* condition has long been debated (for critical review articles, see Hernández *et al*., 2009; Agati *et al*., 2012, 2020), owing to early observations of their almost exclusive location in the vacuoles of epidermal cells (Hrazdina *et al*., 1982; Caldwell *et al*., 1983; Hutzler *et al*., 1998). Instead, flavonoids occur in the vacuoles, the cytoplasm, including the chloroplasts, and the nuclei of parenchymatic cells (Fig. 2; Polster *et al*., 2006; Agati *et al*., 2007, 2009, 2012; Böttner *et al*., 2021) in significantly larger amounts than in the epidermal tissues (Gori *et al*., 2021; Fig. 3). This makes flavonoids ideal for fine‐tuning the ROS concentration in different subcellular compartments, as widely reported in several species (Ferreres *et al*., 2011; Muhlemann *et al*., 2018; Chapman *et al*., 2019; Agati *et al*., 2020; Singh *et al*., 2021; Cerqueira *et al*., 2023). Agati *et al*. (2007) provided conclusive evidence that chloroplast‐located dihydroxy flavonoids (Fig. 2) efficiently quenched singlet oxygen generated by a large excess of photosynthetically active radiation. Flavonols distributed in the cytoplasm and the nuclei of stomata guard cells effectively scavenge H_2_O_2_ (Watkins *et al*., 2014, 2017, see the next section for details). Flavonoids' ability to scavenge ROS may be especially advantageous in plants dealing with multiple environmental stresses, such as when solar irradiance causes severe light stress (Fini *et al*., 2011; Tattini *et al*., 2015). It is known that plants experience severe photooxidative stress, on a daily and seasonal basis, when light irradiance vastly exceeds that usable for photosynthesis, as occurs during the central hours of the day. Light excess is often accompanied by high temperature and vapor pressure deficit, consequently driving stomata closure. The resulting midday depression of photosynthesis, which results in huge ROS production, is further enhanced due to excess light‐ and heat‐induced reduction in the activity of photosynthetic enzymes (Bagley *et al*., 2015; Moore *et al*., 2021). There is evidence that the activity of antioxidant enzymes may fall significantly during the central hours of the day, mostly due to the negative effect of high air temperature (Peltzer & Polle, 2001; Lu *et al*., 2008; Tattini *et al*., 2015; Soengas *et al*., 2018), further enhancing photooxidative stress. The large diurnal variations in flavonoid content recently reported in a range of species, with higher concentrations detected in the midday hours (Barnes *et al*., 2008, 2016; Gori *et al*., 2021), equip plants with not only an effective shield against the penetration of higher levels of UV‐B but also with a more efficient ROS‐scavenging system. We have recently provided evidence that the morning‐to‐midday increase in flavonoid content observed at the whole‐leaf level, almost exclusively involves sub‐epidermal tissues and dihydroxy flavonoids (Gori *et al*., 2021). This is consistent with the common observation that flavonoids with modest ROS‐scavenger capacities respond poorly to light stress and to a variety of other abiotic stimuli (Agati *et al*., 2012; Fig. 3).

While flavonoids have been reported to effectively counter oxidative stress of different origins in a wide range of angiosperms (Agati *et al*., 2020), there is no conclusive evidence for this role in the bryophyte lineages. Stafford (1991) speculated that a fledgling flavonoid metabolism was unlikely to provide flavonoid concentrations suitable for efficient ROS scavenging. However, it is worth noting that flavonoid concentrations in the low μM range are sufficient to effectively counter the oxidative stress, and the extant bryophyte lineage accumulate appreciable concentrations of flavonoids (high nmol to low μmol g^−1^ DW, Albert *et al*., 2018; Liu *et al*., 2022). A recent study has shown that the DELLA TF promotes the exclusive biosynthesis of luteolin 7‐*O*‐glucuronide in *Marchantia polymorpha* and enhances its tolerance to oxidative stress induced by methyl viologen (which mostly generates superoxide anion and hydroxyl radical, Hernández‐García *et al*., 2021). The increase in luteolin 7‐*O* to apigenin 7‐*O*‐glucuronide in UV‐B‐treated *M. polymorpha* also poses an antioxidant role of flavonoids in UV photoprotection (Markham *et al*., 1998, see the next section). This supports the hypothesis of the effective antioxidant role of flavonoids during the evolution of land plant lineages challenged by a wide range of environmental injuries.

## Flavonoids in UV photoprotection: a primary antioxidant function?

There is vast, relatively old, literature supporting the idea that an increase in UV, particularly UV‐B irradiance, was the primary driver for the rise of flavonoid metabolism when plants moved from freshwater to colonize land, which is consistent with the notion that UV‐B radiation greatly enhances flavonoid biosynthesis (Wellmann, 1976; Robberecht & Caldwell, 1978; Caldwell, 1979). It has been inferred that the accumulation of flavonoids in land plants is to primarily equip these plants with an efficient shield against the penetration of the shortest wavelengths of solar radiation. Nonetheless, a very recent UV‐omics investigation indicates that UV radiation likely plays a secondary role compared with water availability during plant terrestrialization (for a review, see Martínez‐Abaigar & Núñez‐Olivera, 2022). In other words, while the biosynthesis of protective sunscreens is an ancestral molecular adaptation of land plants (Rensing, 2018), this does not necessarily favor a primary UV‐B absorbing function of flavonoids in the photoprotection systems of different land plant lineages, including the bryophytes (Agati & Tattini, 2010). Even though early lineages of land plants did experience an increase in UV‐B irradiance when moving from freshwater, it is worth noting that all flavonoids have a relative minimum absorbance at the UV‐B portion (280–315 nm), while maximally absorbing at the UV‐A region of the solar spectrum (usually in the range 330–365 nm; Fig. 1; Agati *et al*., 2009, 2013). This leads to the hypothesis that flavonoids are unlikely to fulfill a primary UV‐B screening function in land plants of varying complexity (Cockell & Knowland, 1999). It is a prerequisite for a metabolite to serve a primary screening function in the overlap between its absorbance spectrum and the light spectrum responsible for its biosynthesis. The biosynthesis of acyl flavonoids, which absorb effectively over the entire range of solar UV wavelengths (Fischbach *et al*., 1999) is a derived trait of land plants, and it is limited to a few species (Tohge *et al*., 2016; Alseekh *et al*., 2020; Wen *et al*., 2020). For instance, we have reported unusual mono‐ and di‐coumaroyl derivatives of Kae 3‐*O*‐glucoside, with outstanding capacity to absorb effectively over the entire solar UV spectrum, in the cell walls of stellate trichomes in leaves of *Cistus salvifolius* (a shrub inhabiting most unfavorable areas of Mediterranean basin, Saracini *et al*., 2005; Tattini *et al*., 2007).

Furthermore, we observe that hydroxycinnamic acid derivatives (HCA), whose concentrations are comparable to those of flavonoids under low UV‐B radiation, are almost unresponsive to increasing UV‐B fluence (Burchard *et al*., 2000; Tattini *et al*., 2000, 2004; Fig. 3). This finding, which conforms to the general observation of UV‐B‐induced increase in flavonoids to HCAs ratio (Agati & Tattini, 2010; Fig. 3), offers conclusive support to the idea of a relatively minor role of flavonoids as UV‐B absorbers in UV‐B photoprotection. HCAs display the greatest absorption capacity over the UV‐B portion of the solar spectrum among the phenylpropanoid pool synthesized by most taxa (Agati *et al*., 2013; Neugart *et al*., 2014; Fig. 1). We note that HCAs distributed on the cuticle matrix, in both the walls and the vacuole of epidermal cells, may effectively limit the entry of UV‐B photons in the leaf, when present in constitutively (i.e. in tissue exposed to low fluence of UV‐B irradiance) high concentrations (Schnitzler *et al*., 1996; Clarke & Robinson, 2008; González Moreno *et al*., 2022). The presence of cuticular HCAs may represent an ancestral mechanism for efficient energy dissipation (Renault *et al*., 2017), based on the observation that the level of cuticular HCA (mainly *p*‐coumaric and ferulic acids) of most bryophytes largely exceed that of the majority of angiosperms (González Moreno *et al*., 2022). The functional significance of HCAs, especially those associated with the cuticle and the epidermal cell walls, in UV‐B photoprotection, has been often underestimated (Mazza *et al*., 2000; Kolb *et al*., 2001; Fabón *et al*., 2010; Monforte *et al*., 2018). However, the matter is of interest, especially when examining the photoprotection mechanisms of land plants at a low degree of body complexity (Renault *et al*., 2017).

Recent evidence of highly conserved mechanisms for sensing and signaling UV‐B radiation in the liverwort *M. polymorpha*, the moss *Physcomitrella patens*, and the flowering plant *A. thaliana* is of interest and conforms to the notion that the UVR8‐signaling pathway has already originated during the movement of plants from the deeper sea to shallow water (Han *et al*., 2019). All the species use the UVR8 photoreceptor and the b‐ZIP TF HY5, a master regulator of light signaling and photomorphogenesis, to acclimate to changes in UV‐B wavelengths (Albert *et al*., 2018; Soriano *et al*., 2018; Podolec *et al*., 2021). Moreover, UV‐B radiation similarly changes the flavonoid pool in both bryophytes and angiosperms, since only the biosynthesis of dihydroxy flavones and flavonols is stimulated by UV‐B radiation (Markham *et al*., 1998; Agati & Tattini, 2010; Wolf *et al*., 2010; Agati *et al*., 2012; Fig. 3). It has been therefore inferred that flavonoids are more involved in countering the photooxidative stress generated by UV‐B radiation, through their ROS‐scavenging capacity, than in avoiding photooxidative stress by acting as sunscreens (Ryan *et al*., 2001; Agati *et al*., 2012; Emiliani *et al*., 2013; Dadras *et al*., 2023b). As a corollary, this offers additional support to early views that high UV‐B irradiance is sensed as an oxidative stress (Landry *et al*., 1995; Jenkins, 2009), just as occurs when plants experience a wide array of abiotic and biotic stressors. Consistently, the very same effective antioxidant flavonoids accumulate to a similar extent in response to high visible or UV‐B radiation in a range of species (Agati *et al*., 2009, 2011; Siipola *et al*., 2016; Albert *et al*., 2018; Taulavuori *et al*., 2018; Zhang *et al*., 2018; Falcone Ferreyra *et al*., 2021). The antioxidant role of flavonoids due to high light intensity may well explain why surface organs such as glandular trichomes, which are autonomous in phenylpropanoid biosynthesis, preferentially accumulate dihydroxy flavonoids at the expense of HCAs in sun‐adapted *Phillyrea latifolia* leaves (Tattini *et al*., 2000; Agati *et al*., 2002; Fig. 2). It is additionally consistent with the primary ROS‐scavenging functions recently attributed to Que 3‐*O*‐rutinoside in glandular trichomes of tomato (Sugimoto *et al*., 2022).

We suggest that following the diversification and efficiency of flavonoid metabolism, which led to the sequential production of flavones, flavonols, and anthocyanins (Li *et al*., 2020) coupled with a versatile transport system, plants had a vast arsenal of metabolites available, capable of limiting the generation (avoidance through light‐screening) and allowing the scavenging of ROS once they are formed. This enabled plants to reverse efficiently photooxidative stress of increasing severity, allowing their successful adaptation in more challenging habitats (Pollastri & Tattini, 2011; Dos Santos Nascimento & Tattini, 2022).

## Flavonoids as signaling molecules: a robust ‘antioxidant‐dependent’ function

The notion that flavonoids act as signaling metabolites has been widely reported in animal cells, and this ability is primarily responsible for the health benefits usually attributed to flavonoids (Williams *et al*., 2004). The capacity of flavonoids to modulate the activity of a range of proteins that may act as downstream components in diverse signaling pathways (mostly of oxidative nature) has been explored to a lesser extent in plants, especially in aboveground organs (Taylor & Grotewold, 2005; Peer & Murphy, 2006; Brunetti *et al*., 2018, 2019; Daryanavard *et al*., 2023). Nonetheless, Helen Stafford proposed, three decades ago, that flavonoids had key functions as internal physiological regulators and chemical messengers, rather than acting as UV‐screening pigments during the colonization of land by plants (Stafford, 1991). She speculated indeed that: (1) a still‐evolving flavonoid metabolism combined with an undeveloped transport system is unlikely to furnish the vacuolar compartment with flavonoid concentrations sufficient to allow optimal UV‐B screening in early land plants; and (2) a primary UV‐screening role does not fit with the extraordinary degree of glycosylation of the flavonoid backbone observed in most plant species. On the contrary, low amounts of flavonoids, which Stafford hypothesized as having been likely synthesized by the first land plants, should have been sufficient to modulate auxin signaling, by acting on both its transport and degradation. Flavonoids had been identified as endogenous regulators of phytochrome‐induced asymmetrical auxin (IAA) distribution, through their ability to modulate the activity of IAA oxidase, in early, seminal experiments conducted at Galston's Lab at Yale University and by Stafford at Reed College in Portland (Furuya *et al*., 1962; Furuya & Thomas, 1964; Bottomley *et al*., 1965; Stafford, 1965). Notably, both low red light and white light supplementation promoted asymmetrical IAA distribution, without affecting Kae glycosides biosynthesis, while strongly inducing Que derivatives biosynthesis in *Pisum sativum* (Bottomley *et al*., 1965). In the same species, Kae derivatives were observed to act as cofactors of IAA oxidase, while Que derivatives successfully hindered the enzyme activity (Furuya *et al*., 1962; Galston, 1969). At the time of Stafford's hypothesis, there was additional evidence of antioxidant flavonoids being also most effective in modulating IAA efflux, based on their ability to inhibit the binding of the synthetic auxin transport inhibitor *N*‐1‐naphthylphthalamic acid (NPA) to a plasma membrane protein (Jacobs & Rubery, 1988). As flavonoids modulate IAA movement and local auxin concentrations at extremely low concentration ranges (from nM to low μM), Stafford speculated this was the ancestral role of flavonoids during plant terrestrialization (Stafford, 1991). Furthermore, she argued that flavonoids might serve these functions in the cytoplasm, near the site of their biosynthesis, that is, the cytoplasmic face of the ER. This argumentation received support later when ancestral IAA auxin efflux PIN proteins, such as the short‐chain PIN5 and PIN8, were discovered to be localized at the ER (Mravec *et al*., 2009; Viaene *et al*., 2014; Ung *et al*., 2022). Incidentally, ER is also the site of IAA biosynthesis (Kriechbaumer *et al*., 2017; Brunetti *et al*., 2018).

There is evidence of plasma membrane‐associated PIN trafficking and polarization mechanisms in *M. polymorpha* and *P. patens* (Skokan *et al*., 2019; Tang *et al*., 2024), and auxin has been reported to influence cell growth and differentiation in both bryophytes (Flores‐Sandoval *et al*., 2024). These findings support Stafford's opinion of an ancestral role of flavonoids as modulators of intra‐ and intercellular IAA movement. We have also hypothesized that flavonoids served a major function as chemical messengers during plant terrestrialization (Brunetti *et al*., 2018), but this matter is far from being fully elucidated, as we discuss below.

The role of flavonoids as chemical messengers has been widely reported for the growth of belowground organs in angiosperms (Hassan & Mathesius, 2012; Ng *et al*., 2020; Ghitti *et al*., 2022), such as in the arbuscular mycorrhizal (AM) association. The effects of flavonoids on AM result from their ability to modulate both local IAA gradients and the level of downstream components of the auxin signaling pathway, as occurs during nodulation (Zhang *et al*., 2009; Abdel‐Lateif *et al*., 2013). The finding that flavonoid aglycones, which are usually exuded by roots, are more effective in promoting AM compared with corresponding glycosylated forms (Zhang *et al*., 2009; Tian *et al*., 2021; Kumar *et al*., 2024), adds further support to the idea that the multifunctionality of flavonoids relates with their antioxidant character. AM association was an event of crucial significance for the adaptability of rootless bryophytes in water‐ and nutrient‐depleted terrestrial habitats (for recent reviews, see Dos Santos Nascimento & Tattini, 2022; Gille *et al*., 2024; Martin & van der Heijden, 2024). Although the putative role of flavonoids in AM association in bryophytes is an attractive suggestion, the strong relationship between flavonoids and auxin observed in angiosperms needs conclusive support in bryophytes. Nonetheless, flavonoids have been recently reported to block auxin transport and inhibit auxin response, thus contributing to 2D‐3D transition in *P. patens* (Moody *et al*., 2021). There is also evidence that SHORT‐LEAF, a member of the Tandem direct repeat‐containing (TDR) proteins regulates gametophore development in *P. pat*ens by mediating the auxin distribution pattern through its strong influence on flavonoid biosynthesis (Palit *et al*., 2024). These findings are remarkable and open the possibility of a putative role of flavonoids as modulators of auxin response and signaling in bryophytes.

The physicochemical features, especially the presence of the catechol group in the B‐ring, confer flavonoids (and other polyphenols) the potential to scavenge ROS and interact with a range of macromolecules as well (Pollastri & Tattini, 2011). For instance, flavonoids may inhibit the activities of a wide array of proteins, including protein kinases by strongly competing with their ATP‐binding sites (structural similarity), as well as acting at the ATP noncompetitive binding site through the formation of both hydrogen bonds and van der Waals interactions (Barron *et al*., 2002; Bode & Dong, 2013). There is compelling evidence that the 3′‐OH group as seen in dihydroxy flavones and flavonols is pivotal for hydrogen bonds with protein kinase backbone amide groups (for a review, see Hou & Kumamoto, 2010). This conforms to the observation that Que and luteolin are more active than Kae and apigenin, respectively, in inhibiting the activities of a range of tyrosine kinases (Chin *et al*., 2013; Alizadeh & Ebrahimzadeh, 2022). There is consensus that these features are significantly more important than the conventional hydrogen‐donating capacity (antioxidant role *sensu stricto*) to explain the effects of flavonoids in the modulation of human cell growth and metabolism (Hou & Kumamoto, 2010; Gu *et al*., 2019). Flavonoids can regulate and modulate the activities of a wide range of proteins in plant cells, including but not limited to protein kinases. For instance, flavonoids inhibit the activity of PIDs, which are serine/threonine kinases that phosphorylate the PIN, IAA efflux carriers (Henrichs *et al*., 2012; Adamowski & Friml, 2015), thus determining their asymmetrical distribution at the plasma membrane, and hence the intercellular IAA fluxes, the well‐known polar IAA transport (PAT). However, flavonoids may also modulate the activities of several ATP‐binding cassette B subfamily (ABCB)‐type IAA transporters (multidrug resistance (MDR) P‐glycoproteins, Blakeslee *et al*., 2005) through bifunctional interactions at both the vicinal ATP‐binding site and the steroid‐interacting region within the protein cytosolic domain (Conseil *et al*., 1998; Ferreira *et al*., 2015). In turn, flavonoids could synergistically inhibit both PIN‐ and ABCB‐based major IAA streams (Mellor *et al*., 2022), through direct association with PINs (Teale *et al*., 2020; Kurepa *et al*., 2023). Indeed, the synthetic inhibitor of IAA transport NPA was shown to lead to conformational perturbation in PIN and hence to decreases in PIN activity (Abas *et al*., 2021). It is not surprising that the antioxidant dihydroxy flavonoids, particularly the flavonol Que, display the greatest inhibitory effect on the activities of PIN and MDR P‐glycoproteins proteins (Mohana *et al*., 2016), and hence in determining IAA gradients in different tissues and cells (Peer & Murphy, 2006, 2007; Michniewicz *et al*., 2007; Bailly *et al*., 2008; Adamowski & Friml, 2015). This may well explain the term ‘developmental regulators’, coined for flavonols by Taylor & Grotewold (2005), a robust function of these molecules in both plants and animals.

We observe that flavonoids may influence IAA gradients in shoots and roots not only by modifying hormone transport at the organ, tissue, cellular, and subcellular levels, but also by influencing IAA catabolism. Early research established that some flavonoids block IAA oxidase (Furuya *et al*., 1962; Bottomley *et al*., 1966), a peroxidase for which flavonoids display strong affinity, as is also the case for vacuolar peroxidases that use flavonoids as preferential substrates to detoxify hydrogen peroxide (H_2_O_2_, Yamasaki *et al*., 1997). This has strong similarities with the mechanisms through which flavonoids inhibit IAA oxidase activity, that is, by serving as preferential substrates compared to IAA for IAA oxidase, and by scavenging H_2_O_2_ generated during early steps of auxin oxidation (Galston *et al*., 1950; Mathesius, 2001). It is not surprising, therefore, that Que and its derivatives are much more potent inhibitors of IAA oxidase than the corresponding Kae‐derived compounds, these last behaving indeed as cofactors of IAA oxidase at certain concentrations (Furuya *et al*., 1962; Bottomley *et al*., 1966). The largely different action of Que and Kae derivatives on IAA oxidase activity may be in part explained by the capacity of Que, but not of Kae derivatives, to chelate Mn (II), a well‐known cofactor of IAA oxidase (Morgan *et al*., 1966). The ability of dihydroxy flavonoids to chelate transition metal ions (De Souza & De Giovani, 2004) has also been used to explain their ability to prevent irreversible oxidative damage in plant nuclei. Dihydroxy flavonoids may efficiently chelate Fe(II)‐ions involved in the Fenton reaction (Fe(II) + H_2_O_2_ → Fe(III) + OH*), thus limiting the formation of hydroxyl radical (OH*) (Agati *et al*., 2012). Recent findings suggest that the major route through which IAA is oxidized in early and modern land plants is by the action of DIOXYGENASE for AUXIN OXIDATION1 protein (DAO1, Zhang *et al*., 2016), a member of the 2‐oxoglutarate and Fe(II)‐dependent (2OG Fe(II)) oxygenase superfamily. Interestingly, an *Arabidopsis* mutant overaccumulating the antioxidant flavonol Que displayed the lowest level of ox‐IAA (Peer *et al*., 2013), likely due to the effective inhibition of DAO activity and scavenging of ROS (Zhang & Peer, 2017). The strong inhibitory effect of antioxidant flavonoids on the activity of proteins regulating IAA‐oxidation is suggested as being of greater significance than their modulation of inter‐ and intra‐cellular auxin movement in determining auxin gradients at cellular and subcellular levels and, hence, in regulating plant growth (Zhang & Peer, 2017).

Overall, this evidence implies that flavonoids play a critical role in modulating the auxin‐signaling network beyond influencing the distribution of IAA at both inter‐ and intra‐cellular levels. Furthermore, relatively recent findings support the notion that flavonoids act as components of a regulatory circuit of the auxin‐signaling pathway. Grunewald *et al*. (2012) have shown that IAA enhances the synthesis of Que derivatives, by acting on the WRKY23 TF and, in turn, Que may fine‐tune IAA distribution, in a PIN‐independent manner. The auxin–flavonol relationship is strong (Blilou *et al*., 2005; Lewis *et al*., 2011) and very recent findings provide conclusive evidence that the IAA repressor IAA17.1, a repressor of early IAA response genes, together with the heat shock protein HSFA5a, promote flavonol biosynthesis and decrease ROS accumulation in salt‐treated roots of *Populus tomentosa* (Song *et al*., 2024).

There is also recent evidence of a robust relationship between flavonols and the abscisic acid (ABA)‐signaling pathway (Gao *et al*., 2021; Segarra‐Medina *et al*., 2023), which may have contributed greatly to the adaptation of plants to the harsh terrestrial habitat (Brunetti *et al*., 2019). The high integration of ABA‐ and light signaling, which occurs at the level of primary signaling components, such as the bZIP TFs ABA Insensitive 5 (ABI5) and HY5 (Chen *et al*., 2008), may well explain the ABA‐induced activation of flavonol biosynthesis, especially of quercetin, in a vast range of species (Berli *et al*., 2010; Alonso *et al*., 2016; Song *et al*., 2022; Castro‐Cegrí *et al*., 2023). It is noted that the crosstalk between ABA and light signaling is an ancient and robust trait of terrestrial plants as the structure and function of HY5 and ABI5 are conserved among early and current‐day land plants (Komatsu *et al*., 2013; Gangappa & Botto, 2016). Flavonols, in turn, regulate the ABA signaling, acting at the level of downstream network components, such as H_2_O_2_ and MAPKs (Brunetti *et al*., 2019). Studies conducted at Gloria Muday's Lab have conclusively shown that flavonols, accumulated (and likely synthesized) in the cytoplasm and nucleus of stomata guard cells, antagonize the closure of stomata by greatly decreasing the levels of H_2_O_2_, a well‐known downstream messenger of the ABA signaling network (Watkins *et al*., 2014, 2017). However, it cannot be excluded that flavonols additionally inhibit the activity of MAPKs that operate downstream of H_2_O_2_ to induce stomata closure (Jammes *et al*., 2009; De Zelicourt *et al*., 2016; Brunetti *et al*., 2019).

## Conclusions: not all flavonoids are equally multifunctional

The functional significance of the diversity and complexity of specialized metabolism has been focused mostly on plant–herbivore interactions and based upon the notions that: (1) most SMs synthesized within specific pathways have low biological activity; and (2) the deployment of a mixture of SMs provides functional synergisms and evolutionary stability (Firn & Jones, 2000; Steppuhun & Baldwin, 2008; Heiling *et al*., 2022; Blanchard & Holeski, 2024).

Consequently, the extraordinary chemical diversity within the flavonoid class, caused by the vast range of glycosylation and substitution patterns of the C6‐C3‐C6 skeleton, complicates a deterministic estimation of their multifunctionality. As previously stated, flavonoids differ significantly in antioxidant capacity, especially when considering the forms found in plant cells. Monohydroxy flavonoid derivatives, for example glycosides of apigenin and Kae, are poor antioxidants (Fig. 1), and their putative effects in an *in planta* condition have been erroneously inferred from studies conducted *in vitro* or *ex‐vivo* using flavonoid aglycones in too many instances (Williamson, 2002). While studies involving flavonoid aglycones may reveal the functions of distinct flavonoid classes in belowground processes (e.g. lateral root emergence; symbiotic nodulation and/or mycorrhizal association Zhang *et al*., 2009; Chapman & Muday, 2021), this is not the case for aboveground organs, which often accumulate flavonoid glycosides in their tissues. Once again, we emphasize that Que 3‐*O*‐glucoside has a lower antioxidant capacity than Que, but has a higher ROS‐scavenging ability than Kae. The antioxidant capacity of Kae 3‐*O*‐glucoside is indeed negligible in a concentration range consistent with its solubility in the aqueous cellular milieu (Fig. 1).

Accordingly, monohydroxy flavones and flavonols have significantly lesser multifunctional potential than their dihydroxy counterparts. It may not be a mere coincidence that in plants exposed to a variety of environmental stresses, including the increase in UV‐B or visible light irradiance, the biosynthesis of antioxidant flavonoids is activated, while the monohydroxy flavonoid pool remains unchanged (for extensive reviews see Agati & Tattini, 2010; Agati *et al*., 2012, 2020; Fig. 3). Data here reported support flavonoids' key activities in both preventing irreversible stress‐induced oxidative damage and modulating different oxidative stress‐induced signaling pathways. Flavonoids tune both ROS levels and the activity of downstream components of oxidative signaling pathways, such as a wide range of protein kinases, in plants and animals. The antioxidant function of flavonoids is, therefore, robust and strongly tied to the plant's ability to evolve (i.e. evolvability, *sensu* Lesne, 2008; Wagner, 2011) in an ever‐changing terrestrial habitat.

In fact, antioxidant flavonoids play a role in stress‐induced morphogenic responses (SIMR), a typical feature of plants exposed to a wide range of stresses (Jansen, 2002; Potters *et al*., 2007), which are, indeed, strongly dependent on ROS (and IAA) signals (Gayomba & Muday, 2020; Martin *et al*., 2022). Flavonoids regulate the auxin‐signaling pathway by severely reducing the activity of proteins that regulate IAA‐oxidation while determining IAA gradients by acting on proteins that escort IAA at intra‐ and intercellular levels. Consistently, flavonoids have been recognized as modulating plant development (reviewed recently in Daryanavard *et al*., 2023), particularly root growth and architecture (Mathesius, 2018; Gayomba & Muday, 2020). Studies examining the involvement of flavonoids in the development of aboveground organs, such as shoot architecture, have yielded conflicting results (Beveridge *et al*., 2007; Buer & Djordjevic, 2009; Buer *et al*., 2013; Fraser *et al*., 2017). This is because most research has been conducted under growth conditions different enough from those often experienced by plants concomitantly facing multiple stressors in their natural solar irradiation when SIMR truly makes sense (Robson *et al*., 2015). For example, high levels of sunlight and UV‐B stimulate or inhibit IAA biosynthesis and signaling, respectively (Hersch *et al*., 2012; Hayes *et al*., 2014; Huq, 2018), whereas both light regimes stimulate the biosynthesis of antioxidant flavonoids (Agati *et al*., 2020). In *Arabidopsis*, a high light‐induced increase in IAA biosynthesis also triggers the biosynthesis of flavonols, particularly of Que (Lewis *et al*., 2011; Grunewald *et al*., 2012). In turn, Que may attenuate local auxin signaling, thus inhibiting apical dominance, as typically occurs in UV‐B‐treated plants under natural conditions (Hayes *et al*., 2014; Robson *et al*., 2015). The mutual regulation of auxin biosynthesis/signaling and flavonoids usually observed in angiosperms is still lacking to be properly described in bryophytes, but very recent studies open new perspectives on this intriguing matter (Moody *et al*., 2021; Palit *et al*., 2024).

The functional significance of the regulatory roles of flavonols on the ABA signaling network has not yet received enough attention, despite the fact they have the potential to significantly regulate the gas exchange performance of plants facing multiple environmental pressures associated with rapid climate change, such as a combination of transient heat waves and rainfall scarcity in high light‐stressed habitats. However, the matter is of primary significance for the ecology of plants with highly diverse complexity.

Overall, we have shown that while flavonoids with varying physicochemical properties have similar abilities to absorb UV radiation and repel herbivores, they differ greatly in their ability to scavenge ROS and hence to modulate both hormone and oxidative signaling pathways. We have provided conclusive evidence that these antioxidant‐related properties, coupled with the distribution in different tissues and cellular compartments, confer only to antioxidant flavonoids the ability to efficiently serve several functions in plants undergoing changes in cellular homeostasis because of a variety of external stimuli. The observation that the biosynthesis of antioxidant flavonoids is a common response of different land plants lineages when confronted with a range of environmental pressures is remarkable, implying that this might represent an ancient feature of land plants.

## Competing interests

None declared.

## Author contributions

LBSN and MT conceived the structure and wrote the MS. CB and AG performed HPLC analysis of phenylpropanoids and estimated the scavenger ability of individual flavonoids for DPPH radical and superoxide anion. GA and ELP determined the UV‐absorbing capacities of individual phenylpropanoids and performed CLSM analyses. All the authors revised and edited the MS.

## References

[nph20195-bib-0001] Abas L , Kolb M , Stadlmann J , Janacek D , Lukic K , Schwechheimer C , Savanov LA , Mach L , Friml J , Hammes UZ . 2021. Naphthylphthalamic acid associates with and inhibits PIN auxin transporters. Proceedings of the National Academy of Sciences, USA 118: e2020857118. 10.1073/pnas.2020857118 PMC7817115 33443187

[nph20195-bib-0002] Abdel‐Lateif K , Vaissavre V , Gherbi H , Verries C , Meudec E , Perrine‐Walker F , Cheynier V , Svistoonoff S , Frnache C , Bogusz D *et al*. 2013. Silencing of the chalcone synthase gene in *Casuarina glauca* highlights the important role of flavonoids during nodulation. New Phytologist 199: 1012–1021. 23692063 10.1111/nph.12326

[nph20195-bib-0003] Adamowski M , Friml J . 2015. PIN‐dependent auxin transport: action, regulation, and evolution. Plant Cell 27: 20–32. 25604445 10.1105/tpc.114.134874 PMC4330589

[nph20195-bib-0004] Agati G , Azzarello E , Pollastri S , Tattini M . 2012. Flavonoids as antioxidants in plants: location and functional significance. Plant Science 196: 67–76. 23017900 10.1016/j.plantsci.2012.07.014

[nph20195-bib-0005] Agati G , Biricolti S , Guidi L , Ferrini F , Fini A , Tattini M . 2011. The biosynthesis of flavonoids is enhanced similarly by UV radiation and root zone salinity in *L. vulgare* leaves. Journal of Plant Physiology 168: 204–212. 20850892 10.1016/j.jplph.2010.07.016

[nph20195-bib-0006] Agati G , Brunetti C , Di Ferdinando M , Ferrini F , Pollastri S , Tattini M . 2013. Functional roles of flavonoids in photoprotection: new evidence, lessons from the past. Plant Physiology and Biochemistry 72: 35–45. 23583204 10.1016/j.plaphy.2013.03.014

[nph20195-bib-0007] Agati G , Brunetti C , Fini A , Gori A , Guidi L , Landi M , Sebastiani F , Tattini M . 2020. Are flavonoids effective antioxidants in plants? Twenty years of our investigation. Antioxidants 9: 1098. 33182252 10.3390/antiox9111098 PMC7695271

[nph20195-bib-0008] Agati G , Galardi C , Gravano E , Romani A , Tattini M . 2002. Flavonoid distribution in tissues of *Phillyrea latifolia* L. leaves as estimated by microspectrofluorometry and multispectral fluorescence microimaging. Photochemistry and Photobiology 76: 350–360. 12403458 10.1562/0031-8655(2002)076<0350:fditop>2.0.co;2

[nph20195-bib-0009] Agati G , Matteini P , Goti A , Tattini M . 2007. Chloroplast‐located flavonoids can scavenge singlet oxygen. New Phytologist 174: 77–89. 17335499 10.1111/j.1469-8137.2007.01986.x

[nph20195-bib-0010] Agati G , Stefano G , Biricolti S , Tattini M . 2009. Mesophyll distribution of ‘antioxidant’ flavonoid glycosides in *Ligustrum vulgare* leaves under contrasting sunlight irradiance. Annals of Botany 104: 853–861. 19633310 10.1093/aob/mcp177 PMC2749533

[nph20195-bib-0011] Agati G , Tattini M . 2010. Multiple functional roles of flavonoids in photoprotection. New Phytologist 186: 786–793. 20569414 10.1111/j.1469-8137.2010.03269.x

[nph20195-bib-0012] Akhtar TA , Lees HA , Lampi MA , Enstone D , Brain RA , Greenberg BM . 2010. Photosynthetic redox imbalance influences flavonoid biosynthesis in *Lemna gibba* . Plant, Cell & Environment 33: 1205–1219. 10.1111/j.1365-3040.2010.02140.x 20199616

[nph20195-bib-0013] Albert NW , Thrimawithana AH , McGhie TK , Clayton WA , Deroles SC , Schwinn KE , Bowman JL , Jordan BR , Davies KM . 2018. Genetic analysis of the liverwort *Marchantia polymorpha* reveals that R2R3MYB activation of flavonoid production in response to abiotic stress is an ancient character in land plants. New Phytologist 218: 554–566. 29363139 10.1111/nph.15002

[nph20195-bib-0014] Ålenius CM , Vogelmann TC , Bornman JF . 1995. A three‐dimensional representation of the relationship between penetration of u.v.‐B radiation and u.v.‐screening pigments in leaves of *Brassica napus* . New Phytologist 131: 287–302.

[nph20195-bib-0015] Alizadeh SR , Ebrahimzadeh MA . 2022. Quercetin derivatives: drug design, development, and biological activities, a review. European Journal of Medicinal Chemistry 229: 114068. 34971873 10.1016/j.ejmech.2021.114068

[nph20195-bib-0016] Alonso R , Berli FJ , Fontana A , Piccoli P , Bottini R . 2016. Malbec grape (*Vitis vinifera* L.) responses to the environment: berry phenolics as influenced by solar UV‐B, water deficit and sprayed abscisic acid. Plant Physiology and Biochemistry 109: 84–90. 27642694 10.1016/j.plaphy.2016.09.007

[nph20195-bib-0017] Alseekh S , Perez de Souza L , Benina M , Fernie AR . 2020. The style and substance of plant flavonoid decoration; towards defining both structure and function. Phytochemistry 174: 112347. 32203741 10.1016/j.phytochem.2020.112347

[nph20195-bib-0018] Baba SA , Wishwakarma RA , Ashra N . 2017. Functional characterization of *Cs*BGlu12, a β‐glucosidase from *Crocus sativus*, provides insights into its role in abiotic stress through accumulation of antioxidant flavonols. Journal of Biological Chemistry 292: 4700–4713. 28154174 10.1074/jbc.M116.762161 PMC5377784

[nph20195-bib-0019] Babu TS , Akhtar T , Lampi MA , Tripuranthakam S , Dixon DG , Greenberg BM . 2003. Similar stress responses are elicited by copper and ultraviolet radiation in the aquatic plant *Lemna gibba*: implication of reactive oxygen species as common signals. Plant & Cell Physiology 44: 1320–1329. 14701927 10.1093/pcp/pcg160

[nph20195-bib-0020] Babu TS , Tripuranhakam S , Greenberg BM . 2005. Biochemical responses of the aquatic higher plant *Lemna gibba* to a mixture of copper and 1,2‐dihydroxyanthraquinone: synergistic toxicity via reactive oxygen species. Environmental Toxicology & Chemistry 24: 3030–3036. 16445081 10.1897/05-073r.1

[nph20195-bib-0021] Bagley J , Rosenthal DM , Ruiz‐Vera UM , Siebers MH , Kumar P , Ort DR , Bernacchi CJ . 2015. The influence of photosynthetic acclimation to rising CO_2_ and warmer temperatures on leaf and canopy photosynthesis models. Global Biogeochemical Cycles 29: 194–206.

[nph20195-bib-0022] Bailly A , Sovero V , Vincenzetti V , Santelia D , Bartnik D , Koenig BW , Mancuso S , Martinoia E , Geisler M . 2008. Modulation of P‐glycoproteins by auxin transport inhibitors is mediated by interaction with immunophilins. Journal of Biological Chemistry 283: 21817–21826. 18499676 10.1074/jbc.M709655200

[nph20195-bib-0023] Ballaré CL . 2014. Light regulation of plant defense. Annual Review of Plant Biology 65: 335–363. 10.1146/annurev-arplant-050213-040145 24471835

[nph20195-bib-0024] Baratto MC , Tattini M , Galardi C , Pinelli P , Romani A , Visiol F , Basosi R , Pogni R . 2003. Antioxidant activity of galloyl quinic derivatives isolated from *P. lentiscus* leaves. Free Radical Research 37: 405–412. 12747734 10.1080/1071576031000068618

[nph20195-bib-0025] Barnes PW , Tobler MA , Keefover‐Ring K , Flint SD , Barkley AE , Ryels RJ , Lindroth RL . 2016. Rapid modulation of ultraviolet shielding in plants is influenced by solar ultraviolet radiation and linked to alterations in flavonoids. Plant, Cell & Environment 39: 222–230. 10.1111/pce.12609 26177782

[nph20195-bib-0026] Barnes W , Flint SD , Slusser JR , Gao W , Ryel RJ . 2008. Diurnal changes in epidermal UV transmittance of plants in naturally high UV environments. Physiologia Plantarum 133: 363–372. 18346077 10.1111/j.1399-3054.2008.01084.x

[nph20195-bib-0027] Barron D , Di Pietro A , Dumontet C , McIntosh D . 2002. Isoprenoid flavonoids are new leads in the modulation of chemoresistance. Phytochemistry Reviews 1: 325–332.

[nph20195-bib-0028] Berli FJ , Moreno D , Piccoli P , Hespanhol‐Viana L , Silva MF , Bressan‐Smith R , Cavagnaro JB , Bottini R . 2010. Abscisic acid is involved in the response of grape (*Vitis vinifera* L.) cv. Malbec leaf tissues to ultraviolet‐B radiation by enhancing ultraviolet‐absorbing compounds, antioxidant enzymes and membrane sterols. Plant, Cell & Environment 33: 1–10. 10.1111/j.1365-3040.2009.02044.x 19781012

[nph20195-bib-0029] Beveridge CA , Mathesius U , Rose RJ , Gresshoff PM . 2007. Common regulatory themes in meristem development and whole‐plant homeostasis. Current Opinion in Plant Biology 10: 44–51. 17157052 10.1016/j.pbi.2006.11.011

[nph20195-bib-0030] Bian H‐X , Li W , Niu C‐F , Wei W , Hu Y , Han J‐Q , Lu X , Tao J‐J , Jin M , Qin H *et al*. 2019. A class B heat shock factor selected for during soybean domestication contributes to salt tolerance by promoting flavonoid biosynthesis. New Phytologist 225: 268–283. 31400247 10.1111/nph.16104

[nph20195-bib-0031] Blakeslee JJ , Peer WA , Murphy AS . 2005. Auxin transport. Current Opinion in Plant Biology 8: 494–500. 16054428 10.1016/j.pbi.2005.07.014

[nph20195-bib-0032] Blanchard M , Holeski LM . 2024. Consequences and costs of chemical complexity: the evolutionary ecology of direct phytochemical defense against herbivores. International Journal of Plant Sciences 185: 3–14.

[nph20195-bib-0033] Blilou I , Xu J , Wildwater M , Willemsen V , Paponov I , Friml J , Heidstra R , Aida M , Palme K , Scheres B . 2005. The PIN auxin efflux facilitator network controls growth and patterning in Arabidopsis roots. Nature 433: 39–44. 15635403 10.1038/nature03184

[nph20195-bib-0034] Bode AM , Dong Z . 2013. Signal transduction and molecular targets of selected flavonoids. Antioxidants & Redox Signaling 19: 163–180. 23458437 10.1089/ars.2013.5251 PMC3689254

[nph20195-bib-0035] Böttner L , Grabe V , Gablenz S , Böhme N , Appenroth KJ , Gershenzon J , Huber M . 2021. Differential localization of flavonoid glucosides in an aquatic plant implicates different functions under abiotic stress. Plant, Cell & Environment 44: 900–914. 10.1111/pce.13974 33300188

[nph20195-bib-0036] Bottomley W , Smith H , Galston AW . 1965. A phytochrome mediated effect of light on the hydroxylation pattern of flavonoids in *Pisum sativum* var. ‘Alaska’. Nature 207: 1311–1312.5884659

[nph20195-bib-0037] Bottomley W , Smith H , Galston AW . 1966. Flavonoid complexes in *Pisum sativum* – III. The effect of light on the synthesis of kaempferol and quercetin complexes. Phytochemistry 5: 117–123.

[nph20195-bib-0038] Bowles AMC , Bechtold U , Paps J . 2020. The origin of land plants is rooted in two bursts of genomic novelty. Current Biology 30: 530–536. 31956023 10.1016/j.cub.2019.11.090

[nph20195-bib-0039] Bowman JL , Kohchi T , Yamato KT , Jenkins J , Shu S , Ishizaki K , Yamaoka S , Nishihama R , Nakamura Y , Berger F *et al*. 2017. Insights into land plant evolution garnered from the *Marchantia polymorpha* genome. Cell 171: 287–304. 28985561 10.1016/j.cell.2017.09.030

[nph20195-bib-0041] Brunetti C , Fini A , Sebastiani F , Gori A , Tattini M . 2018. Modulation of phytohormone signaling: a primary function of flavonoids in plant–environment interactions. Frontiers in Plant Science 9: 1042. 30079075 10.3389/fpls.2018.01042 PMC6062965

[nph20195-bib-0042] Brunetti C , Sebastiani F , Tattini M . 2019. ABA, flavonols and the evolvability of land plants. Plant Science 280: 448–454. 30824025 10.1016/j.plantsci.2018.12.010

[nph20195-bib-0043] Buer CS , Djordjevic MA . 2009. Architectural phenotypes in the *transparent testa* mutants of *Arabidopsis thaliana* . Journal of Experimental Botany 60: 751–763. 19129166 10.1093/jxb/ern323 PMC2652062

[nph20195-bib-0044] Buer CS , Imin N , Djordjevic MA . 2010. Flavonoids: new roles for old molecules. Journal of Integrative Plant Biology 52: 98–111. 20074144 10.1111/j.1744-7909.2010.00905.x

[nph20195-bib-0045] Buer CS , Kordbacheh F , Truong TT , Hocart CH , Djordjevic MA . 2013. Alteration of flavonoid accumulation patterns in *transparent testa* mutants disturbs auxin transport, gravity responses, and imparts long‐term effects on root and shoot architecture. Planta 238: 171–189. 23624937 10.1007/s00425-013-1883-3

[nph20195-bib-0046] Burchard P , Bilger W , Weissenböck G . 2000. Contribution of hydroxycinnamates and flavonoids to epidermal shielding of UV‐A and UV‐B radiation in developing rye primary leaves as assessed by ultraviolet‐induced chlorophyll fluorescence measurements. Plant, Cell & Environment 23: 1373–1380.

[nph20195-bib-0047] Buschmann H . 2020. Into another dimension: how streptophyte algae gained morphological complexity. Journal of Experimental Botany 71: 3279–3286. 32270175 10.1093/jxb/eraa181

[nph20195-bib-0048] Caldwell MM . 1979. Plant life and ultraviolet radiation: some perspective in the history of the Earth's UV climate. Bioscience 29: 520–525.

[nph20195-bib-0049] Caldwell MM , Robberecht R , Flint SD . 1983. Internal filters: prospects for UV‐acclimation in higher plants. Physiologia Plantarum 58: 445–450.

[nph20195-bib-0050] Cannell N , Emms DM , Hetherington AJ , MacKay J , Kelly S , Dolan L , Sweetlove LG . 2020. Multiple metabolic innovations and losses are associated with major transitions in land plant evolution. Current Biology 30: 1783–1800. 32220326 10.1016/j.cub.2020.02.086

[nph20195-bib-0051] Castro‐Cegrí A , Sierra S , Hidalgo‐Santiago L , Esteban‐Muñoz A , Jamilena M , Garrido D , Palma F . 2023. Postharvest treatment with abscisic acid alleviates chilling injury in zucchini fruit by regulating phenolic metabolism and non‐enzymatic antioxidant system. Antioxidants 12: 211. 36671073 10.3390/antiox12010211 PMC9854589

[nph20195-bib-0052] Cerqueira JVA , de Andrade MT , Rafael DD , Zhu F , Martins SVC , Nunes‐Nesi A , Benedito V , Fernie AR , Zsögön A . 2023. Anthocyanins and reactive oxygen species: a team of rivals regulating plant development? Plant Molecular Biology 112: 213–223. 37351824 10.1007/s11103-023-01362-4 PMC10352431

[nph20195-bib-0053] Chapman JM , Muday GK . 2021. Flavonols modulate lateral root emergence by scavenging reactive oxygen species in *Arabidopsis thaliana* . Journal of Biological Chemistry 296: 100222. 33839683 10.1074/jbc.RA120.014543 PMC7948594

[nph20195-bib-0054] Chapman JR , Muhlemann JK , Gayomba SR , Muday GK . 2019. RBOH‐dependent ROS synthesis and ROS scavenging by plant specialized metabolites to modulate plant development and stress responses. Chemical Research in Toxicology 32: 370–396. 30781949 10.1021/acs.chemrestox.9b00028 PMC6857786

[nph20195-bib-0055] Chen H , Zhang J , Neff MM , Xiong L . 2008. Integration of light and abscisic acid signaling during seed germination and early seedling development. Proceedings of the National Academy of Sciences, USA 105: 4495–4500. 10.1073/pnas.0710778105 PMC2393781 18332440

[nph20195-bib-0056] Cheng S , Xian W , Fu Y , Marin B , Keller J , Wu T , Sun W , Li X , Xu Y , Zhang Y *et al*. 2019. Genomes of subaerial *Zygnematophyceae* provide insights into land plant evolution. Cell 179: 1057–1067. 31730849 10.1016/j.cell.2019.10.019

[nph20195-bib-0057] Chin Y‐W , Kong JY , Han S‐Y . 2013. Flavonoids as receptor tyrosine kinase FLT3 inhibitors. Bioorganic & Medicinal Chemistry Letters 23: 1768–1770. 23411073 10.1016/j.bmcl.2013.01.049

[nph20195-bib-0058] Clarke LJ , Robinson SA . 2008. Cell wall‐bound ultraviolet‐screening compounds explain the high ultraviolet tolerance of the Antarctic moss, *Ceratodon purpureus* . New Phytologist 179: 776–783. 18513223 10.1111/j.1469-8137.2008.02499.x

[nph20195-bib-0060] Close DC , McArthur C . 2002. Rethinking the role of many plant phenolics–protection from photodamage not herbivores? Oikos 99: 166–172.

[nph20195-bib-0061] Cockell CS , Knowland J . 1999. Ultraviolet radiation screening compounds. Biological Reviews 74: 311–345. 10466253 10.1017/s0006323199005356

[nph20195-bib-0062] Conseil G , Baubichon‐Cortay H , Dayan G , Di Pietro AD . 1998. Flavonoids: a class of modulators with bifunctional interactions at vicinal ATP‐ and steroid‐binding sites on mouse P‐glycoprotein. Proceedings of the National Academy of Science, USA 95: 9831–9836. 10.1073/pnas.95.17.9831 PMC21422 9707561

[nph20195-bib-0063] Constabel PC , Yoshida K , Walker V . 2014. Diverse ecological roles of plant tannins: plant defense and beyond. In: Romani A , Lattanzio V , Quideau S , eds. Recent advances in polyphenol research. Hoboken, NJ, USA: John Wiley & Sons, 115–142.

[nph20195-bib-0064] Cooper Driver GA . 1980. The role of flavonoids and related compounds in fern systematics. Bulletin of the Torrey Botanical Club 107: 116–127.

[nph20195-bib-0065] Dadras A , Furst‐Jansen JMR , Darienko T , Krone D , Scholz P , Sun S , Herrfurth C , Rieseberg TP , Irisarri I , Steinkamp R . 2023a. Environmental gradients reveal stress hubs pre‐dating plant terrestrialization. Nature Plants 9: 1419–1438. 37640935 10.1038/s41477-023-01491-0 PMC10505561

[nph20195-bib-0066] Dadras A , Rieseberg TP , Zegers JMS , Irisarri I , de Vries J , de Vries S . 2023b. Accessible versatility underpins the deep evolution of plant specialized metabolism. Phytochemistry Reviews. doi: 10.1007/s11101-023-09863-2. PMC11842411 39991433

[nph20195-bib-0067] Daryanavard H , Postiglione AE , Muhlemann JK , Muday GK . 2023. Flavonols modulate plant development, signaling, and stress responses. Current Opinion in Plant Biology 72: 102350. 36870100 10.1016/j.pbi.2023.102350 PMC10372886

[nph20195-bib-0068] Davies KM , Jibran R , Albert NW , Zhou Y , Schwinn KE . 2021. Conservation and divergence between bryophytes and angiosperms in the biosynthesis and regulation of flavonoid production. In: Reed DJ , Perera de Freitas VA , Quideau S , eds. Recent advances in polyphenol research, vol. 7. Hoboken, NJ, USA: John Wiley & Sons, 227–263.

[nph20195-bib-0069] Davies KM , Jibran R , Zhou Y , Albert NW , Brummell DA , Jordan BR , Bowman JL , Schwinn KE . 2020. The evolution of flavonoid biosynthesis: a bryophyte perspective. Frontiers in Plant Science 11: 7. 32117358 10.3389/fpls.2020.00007 PMC7010833

[nph20195-bib-0070] De Souza RFV , De Giovani WF . 2004. Antioxidant properties of complexes of flavonoids with metal ions. Redox Report 9: 97–104. 15231064 10.1179/135100004225003897

[nph20195-bib-0071] De Zelicourt A , Colcombet J , Hirt H . 2016. The role of MAPK modules and ABA during abiotic stress signaling. Trends in Plant Science 21: 677–685. 27143288 10.1016/j.tplants.2016.04.004

[nph20195-bib-0072] Devireddy AR , Zandalinas SI , Fichman Y , Mittler R . 2021. Integration of reactive oxygen species and hormone signaling during abiotic stress. The Plant Journal 105: 459–476. 33015917 10.1111/tpj.15010

[nph20195-bib-0073] Dietz K‐J , Vogelsang L . 2024. A general concept of quantitative abiotic stress sensing. Trends in Plant Science 29: 319–328. 37591742 10.1016/j.tplants.2023.07.006

[nph20195-bib-0074] Dixon RA , Dickinson AJ . 2024. A century of studying plant secondary metabolism – from “what?” to “where, how, and why?”. Plant Physiology 195: 48–66. 38163637 10.1093/plphys/kiad596 PMC11060662

[nph20195-bib-0075] Donoghue PCJ , Harrison CJ , Paps J , Scheider H . 2021. The evolutionary emergence of land plants. Current Biology 32: R1281–R1298. 10.1016/j.cub.2021.07.038 34637740

[nph20195-bib-0076] Dos Santos Nascimento LB , Tattini M . 2022. Beyond photoprotection: the multifarious roles of flavonoids in plant terrestrialization. International Journal of Molecular Sciences 23: 5284. 35563675 10.3390/ijms23095284 PMC9101737

[nph20195-bib-0077] Durán‐Medina Y , Ruiz‐Cortés BE , Guerrero‐Largo H , Marsch‐Martínez N . 2021. Specialized metabolism and development: an unexpected friendship. Current Opinion in Plant Biology 64: 102142. 34856480 10.1016/j.pbi.2021.102142

[nph20195-bib-0078] Ehlers BK , Berg MP , Staudt M , Holmstrup M , Glasius M , Ellers J , Tomiolo S , Madsen RB , Slotsbo S , Penuelas J . 2020. Plant secondary compounds in soil and their role in belowground species interactions. Trends in Ecology & Evolution 35: 716–730. 32414604 10.1016/j.tree.2020.04.001

[nph20195-bib-0079] Ehrlich PR , Raven PH . 1964. Butterflies and plants: a study in coevolution. Evolution 18: 586–608.

[nph20195-bib-0080] Emiliani J , Grotewold E , Falcone Ferreyra ML , Casati P . 2013. Flavonols protect Arabidopsis plants against UV‐B deleterious effects. Molecular Plant 6: 1376–1379. 23371934 10.1093/mp/sst021

[nph20195-bib-0081] Erb M , Kliebenstein DJ . 2020. Plant secondary metabolites as defenses, regulators, and primary metabolites: the blurred functional trichotomy. Plant Physiology 184: 39–52. 32636341 10.1104/pp.20.00433 PMC7479915

[nph20195-bib-0082] Fabón G , Martinez‐Abaigar J , Tomás R , Núñez‐Olivera E . 2010. Effects of enhanced UV‐B radiation on hydroxycinnamic acid derivatives extracted from different cell compartments in the aquatic liverwort *Jungermannia exsertifolia* subsp. *cordifolia* . Physiologia Plantarum 140: 269–279. 20663084 10.1111/j.1399-3054.2010.01401.x

[nph20195-bib-0083] Falcone Ferreyra ML , Serra P , Casati P . 2021. Recent advances on the roles of flavonoids as plant protective molecules after UV and high light exposure. Physiologia Plantarum 173: 736–749. 34453749 10.1111/ppl.13543

[nph20195-bib-0084] Feller A , Machemer K , Braun EL , Grotewold E . 2011. Evolutionary and comparative analysis of MYB and bHLH plant transcription factors. The Plant Journal 66: 94–116. 21443626 10.1111/j.1365-313X.2010.04459.x

[nph20195-bib-0085] Ferreira A , Pousinho S , Fortuna A , Falcao A , Alves G . 2015. Flavonoid compounds as reversal agents of the P‐glycoprotein‐mediated multidrug resistance: biology, chemistry and pharmacology. Phytochemistry Reviews 14: 233–272.

[nph20195-bib-0086] Ferreres F , Figueiredo R , Bettencourt S , Corqueijeiro I , Oliveira J , Gil‐Izquerdio A , Pereira DM , Valentão P , Andrade PB , Duarte P *et al*. 2011. Identification of phenolic compounds in isolated vacuoles of the medicinal plant *Catharanthus roseus* and their interaction with vacuolar class III peroxidase: an H_2_O_2_ affair? Journal of Experimental Botany 62: 2841–2854. 21357771 10.1093/jxb/erq458

[nph20195-bib-0087] Fini A , Brunetti C , Di Ferdinando M , Ferrini F , Tattini M . 2011. Stress‐induced flavonoid biosynthesis and the antioxidant machinery of plants. Plant Signaling & Behavior 6: 709–711. 21448007 10.4161/psb.6.5.15069 PMC3172844

[nph20195-bib-0088] Firn RD , Jones CG . 2000. The evolution of secondary metabolism – a unifying model. Molecular Microbiology 37: 989–994. 10972818 10.1046/j.1365-2958.2000.02098.x

[nph20195-bib-0089] Fischbach RJ , Kossmann B , Panten H , Steinbrecher R , Heller W , Seidlitz HK , Sandermann H , Hertkorn N , Schnitzler J‐P . 1999. Seasonal accumulation of ultraviolet‐B screening pigments in needles of Norway spruce (*Picea abies* (L.) Karst.). Plant, Cell & Environment 22: 27–37.

[nph20195-bib-0090] Flores‐Sandoval E , Nishihama R , Bowman JL . 2024. Hormonal and genetic control of pluripotency in bryophyte model systems. Current Opinion in Plant Biology 77: 102486. 38041967 10.1016/j.pbi.2023.102486

[nph20195-bib-0091] Fraser DP , Sharma A , Fletcher T , Budge S , Moncrieff C , Dodd AN , Franklin K . 2017. UV‐B antagonises shade avoidance and increases levels of the flavonoid quercetin in coriander (*Coriandrum sativum*). Scientific Reports 7: 17758. 29259256 10.1038/s41598-017-18073-8 PMC5736551

[nph20195-bib-0092] Fürst‐Jansen JMR , de Vries S , de Vries J . 2020. Evo‐physio: on stress responses and the earliest land plants. Journal of Experimental Botany 71: 3254–3269. 31922568 10.1093/jxb/eraa007 PMC7289718

[nph20195-bib-0093] Furuya M , Galston A , Stowe B . 1962. Isolation from peas of co‐factors and inhibitors of indolyl‐3‐acetic acid oxidase. Nature 193: 456–457. 13896010 10.1038/193456a0

[nph20195-bib-0094] Furuya M , Thomas RG . 1964. Flavonoid complexes in *Pisum sativum*. II. Effects of red and far‐red light on biosynthesis of kaempferol complexes and on growth of etiolated plumules. Plant Physiology 39: 634–642. 16655976 10.1104/pp.39.4.634 PMC550138

[nph20195-bib-0095] Galston AW . 1969. Flavonoids and photomorphogenesis in peas. In: Harborne JB , Swain T , eds. Perspectives in phytochemistry. New York, NY, USA: Academic Press, 193–204.

[nph20195-bib-0096] Galston AW , Bonner J , Baker RS . 1950. Flavoprotein and peroxidase as constituents of the indoleacetic acid oxidase of peas. American Journal of Botany 37: 677–678.

[nph20195-bib-0097] Gangappa SN , Botto JF . 2016. The multifaceted roles of HY5 in plant growth and development. Molecular Plant 9: 1353–1365. 27435853 10.1016/j.molp.2016.07.002

[nph20195-bib-0098] Gao G , Lv Z , Zhang G , Li J , Zhang J , He C . 2021. An ABA–flavonoid relationship contributes to the differences in drought resistance between different sea buckthorn subspecies. Tree Physiology 41: 744–755. 33184668 10.1093/treephys/tpaa155

[nph20195-bib-0099] Garagounis C , Delkis N , Papadopoulou KK . 2021. Unraveling the roles of plant specialized metabolites: using synthetic biology to design molecular biosensors. New Phytologist 231: 1338–1352. 33997999 10.1111/nph.17470

[nph20195-bib-0100] Garcia‐Molina A , Pastor V . 2024. Systemic analysis of metabolome reconfiguration in Arabidopsis after abiotic stressors uncovers metabolites that modulate defense against pathogens. Plant Communications 5: 100645. 37403356 10.1016/j.xplc.2023.100645 PMC10811363

[nph20195-bib-0101] Gayomba SR , Muday GK . 2020. Flavonols regulate root hair development by modulating accumulation of reactive oxygen species in the root epidermis. Development 147: dev185819. 32179566 10.1242/dev.185819

[nph20195-bib-0102] Gerhardt KE , Lampi MA , Greenberg BM . 2008. The effects of far‐red light on plant growth and flavonoid accumulation in *Brassica napus* in the presence of ultraviolet B radiation. Photochemistry and Photobiology 84: 1445–1454. 18466203 10.1111/j.1751-1097.2008.00362.x

[nph20195-bib-0103] Ghitti E , Rolli E , Crotti E , Borin S . 2022. Flavonoids are intra‐ and inter‐kingdom modulator signals. Microorganisms 10: 2479. 36557733 10.3390/microorganisms10122479 PMC9781135

[nph20195-bib-0104] Gille CE , Finnegan PM , Hayes PE , Ranathunge K , Burgess TI , de Tombeur F , Migliorini D , Dallongeville P , Glauser G , Lambers H . 2024. Facilitative and competitive interactions between mycorrhizal and non‐mycorrhizal plants in an extremely phosphorus‐impoverished environment: role of ectomycorrhizal fungi and native oomycete pathogens in shaping species coexistence. New Phytologist 242: 1630–1640. 38105548 10.1111/nph.19489

[nph20195-bib-0105] González Moreno A , de Cózar A , Prieto P , Domínguez E , Heredia A . 2022. Radiationless mechanism of UV deactivation by cuticle phenolics in plants. Nature Communications 13: 1786. 10.1038/s41467-022-29460-9 PMC8979964 35379806

[nph20195-bib-0106] Gori A , Brunetti C , dos Santos Nascimento LB , Marino G , Guisi L , Ferrini F , Centritto M , Fini A , Tattini M . 2021. Photoprotective role of photosynthetic and non‐photosynthetic pigments in *Phillyrea latifolia*: is their “antioxidant” function prominent in leaves exposed to severe summer drought? International Journal of Molecular Sciences 22: 8303. 34361067 10.3390/ijms22158303 PMC8347396

[nph20195-bib-0107] Gourlay G , Constabel CP . 2019. Condensed tannins are inducible antioxidants and protect hybrid poplar against oxidative stress. Tree Physiology 39: 345–355. 30917196 10.1093/treephys/tpy143

[nph20195-bib-0108] Grunewald W , De Smet I , Lewis DR , Löfke C , Jansen L , Geominne G , Vanden Bossche R , Karimi M , De Rybel B , Vanholme B . 2012. Transcription factor WRKY23 assists auxin distribution patterns during Arabidopsis root development through local control on flavonol biosynthesis. Proceedings of the National Academy of Sciences, USA 109: 1554–1559. 10.1073/pnas.1121134109 PMC3277162 22307611

[nph20195-bib-0109] Gu C , Stashko MA , Puhl‐Rubio AC , Chakraborty M , Chakraborty A , Frye SV , Pearce KH , Wang X , Shears SB , Wang H . 2019. Inhibition of inositol polyphosphate kinases by quercetin and related flavonoids: a structure‐activity analysis. Journal of Medicinal Chemistry 62: 1443–1454. 30624931 10.1021/acs.jmedchem.8b01593 PMC6467728

[nph20195-bib-0110] Han X , Chang X , Zhang Z , Chen H , He H , Zhong B , Deng XW . 2019. Origin and evolution of core components responsible for monitoring light environment changes during plant terrestrialization. Molecular Plant 12: 847–862. 31009752 10.1016/j.molp.2019.04.006

[nph20195-bib-0111] Hassan S , Mathesius U . 2012. The role of flavonoids in root–rhizosphere signalling: opportunities and challenges for improving plant–microbe interactions. Journal of Experimental Botany 63: 3429–3444. 22213816 10.1093/jxb/err430

[nph20195-bib-0112] Hayes S , Velanis CN , Jenkins GI , Franklin KA . 2014. UV‐B detected by the UVR8 photoreceptor antagonizes auxin signaling and plant shade avoidance. Proceedings of the National Academy of Sciences, USA 111: 11894–11899. 10.1073/pnas.1403052111 PMC4136589 25071218

[nph20195-bib-0113] He H , Van Breusegem F , Mhamdi A . 2018. Redox‐dependent control of nuclear transcription in plants. Journal of Experimental Botany 69: 3359–3372. 29659979 10.1093/jxb/ery130

[nph20195-bib-0114] Heiling S , Li J , Halitschke R , Paetz C , Baldwin IT . 2022. The downside of metabolic diversity: postingestive rearrangements by specialized insects. Proceedings of the National Academy of Sciences, USA 119: e2122808119. 10.1073/pnas.2122808119 PMC9214519 35666864

[nph20195-bib-0115] Heine GF , Hernandez JM , Grotewold E . 2004. Two cysteines in plant R2R3 MYB domains participate in redox‐dependent DNA binding. Journal of Biological Chemistry 279: 37878–37885. 15237103 10.1074/jbc.M405166200

[nph20195-bib-0116] Henrichs S , Wang B , Fukao Y , Zhu J , Charrier L , Bailly A , Oehring SC , Linnert M , Weiward A , Endler A *et al*. 2012. Regulation of ABCB1/PGP1‐catalysed auxin transport by linker phosphorylation. EMBO Journal 31: 2965–2980. 22549467 10.1038/emboj.2012.120 PMC3395086

[nph20195-bib-0117] Hernández I , Alegre A , Van Breusegem F , Munné‐Bosch S . 2009. How relevant are flavonoids as antioxidants in plants? Trends in Plant Science 14: 125–132. 19230744 10.1016/j.tplants.2008.12.003

[nph20195-bib-0118] Hernández‐García J , Serrano‐Mislata A , Inoue K , Vargas‐Chávez C , Esteva‐Bruna D , Arbona V , Yamaoka S , Nishihama R , Kocchi T *et al*. 2021. Coordination between growth and stress responses by DELLA in the liverwort *Marchantia polymorpha* . Current Biology 31: 3678–3686. 34214451 10.1016/j.cub.2021.06.010

[nph20195-bib-0119] Hersch M , Lorrain S , de Wit M , Frankhauser C . 2012. Light intensity modulates the regulatory network of the shade avoidance response in *Arabidopsis* . Proceedings of the National Academy of Sciences, USA 111: 6515–6520. 10.1073/pnas.1320355111 PMC4035961 24733935

[nph20195-bib-0120] Hou D‐X , Kumamoto T . 2010. Flavonoids as protein kinase inhibitors for cancer chemoprevention: direct binding and molecular modeling. Antioxidants & Redox Signaling 13: 691–719. 20070239 10.1089/ars.2009.2816

[nph20195-bib-0121] Hrazdina G , Marx GA , Hoch HC . 1982. Distribution of secondary plant metabolites and their biosynthetic enzymes in pea (*Pisum sativum* L.) leaves: anthocyanins and flavonol glycosides. Plant Physiology 70: 745–748. 16662568 10.1104/pp.70.3.745 PMC1065763

[nph20195-bib-0122] Hu L , Wu Z , Robert C , Erb M . 2021. Soil chemistry determines whether defensive plant secondary metabolites promote or suppress herbivore growth. Proceedings of the National Academy of Sciences, USA 118: e2109602118. 10.1073/pnas.2109602118 PMC8639379 34675080

[nph20195-bib-0123] Huq E . 2018. Direct convergence of light and auxin signaling pathways in Arabidopsis. Molecular Plant 11: 515–517. 29458178 10.1016/j.molp.2018.02.002 PMC5882582

[nph20195-bib-0124] Hutzler P , Fishback R , Heller W , Jungblut TP , Reuber S , Schmitz R , Veit M , Weissenböck G , Schnitzler J‐P . 1998. Tissue localization of phenolic compounds in plants by confocal laser scanning microscopy. Journal of Experimental Botany 49: 953–965.

[nph20195-bib-0125] Imran QM , Hussain A , Lee S‐U , Mun B‐G , Falak N , Loake GJ , Yun B‐W . 2018. Transcriptome profile of NO‐induced Arabidopsis transcription factor genes suggests their putative regulatory role in multiple biological processes. Scientific Reports 8: 771. 29335449 10.1038/s41598-017-18850-5 PMC5768701

[nph20195-bib-0126] Izaguiree MM , Scopel AL , Baldwin IT , Ballaré CL . 2003. Convergent responses to stress. Solar ultraviolet‐B radiation and *Manduca sexta* herbivory elicit overlapping transcriptional responses in field‐grown plants of *Nicotiana longiflora* . Plant Physiology 132: 1755–1767. 12913133 10.1104/pp.103.024323 PMC181263

[nph20195-bib-0127] Izhaki K . 2002. Emodin – a secondary metabolite with multiple ecological functions in higher plants. New Phytologist 155: 205–217.

[nph20195-bib-0128] Jacobs M , Rubery PH . 1988. Naturally occurring auxin transport regulators. Science 241: 346–349. 17734864 10.1126/science.241.4863.346

[nph20195-bib-0129] Jammes F , Song C , Shin D , Kwak JM . 2009. MAP kinases *MPK9* and *MPK12* are preferentially expressed in guard cells and positively regulate ROS‐mediated ABA signaling. Proceedings of the National Academy of Sciences, USA 106: 20520–20525. 10.1073/pnas.0907205106 PMC2776606 19910530

[nph20195-bib-0130] Jansen MAK . 2002. Ultraviolet‐B radiation effects on plants: induction of morphogenic responses. Physiologia Plantarum 116: 423–429.

[nph20195-bib-0131] Jenkins GI . 2009. Signal transduction in responses to UV‐B radiation. Annual Review of Plant Biology 60: 407–431. 10.1146/annurev.arplant.59.032607.092953 19400728

[nph20195-bib-0132] Jiang C‐K , Rao G‐Y . 2020. Insights into the diversification and evolution of R2R3‐MYB transcription factors in plants. Plant Physiology 183: 637–655. 32291329 10.1104/pp.19.01082 PMC7271803

[nph20195-bib-0133] Jones CG , Firn RD . 1991. On the evolution of plant secondary diversity. Philosophical Transactions of the Royal Society of London. Series B: Biological Sciences 333: 273–280.

[nph20195-bib-0134] Keeling CI , Dullat HK , Yuen M , Ralph SG , Jancsik S , Bohlmann J . 2010. Identification and functional characterization of monofunctional *ent*‐copalyl diphosphate and *ent*‐kaurene synthases in white spruce reveal different patterns for diterpene synthase evolution for primary and secondary metabolism in gymnosperms. Plant Physiology 152: 1197–1208. 20044448 10.1104/pp.109.151456 PMC2832265

[nph20195-bib-0135] Ketudat Cairns JR , Mahong B , Baiya S , Jeon J‐S . 2015. β‐Glucosidases: multitasking, moonlighting or simply misunderstood? Plant Science 241: 246–259. 26706075 10.1016/j.plantsci.2015.10.014

[nph20195-bib-0136] Kim RJ , Lee SB , Pandey G , Suh MC . 2022. Functional conservation of an AP2/ERF transcription factor in cuticle formation suggests an important role in the terrestrialization of early land plants. Journal of Experimental Botany 73: 7450–7466. 36112045 10.1093/jxb/erac360

[nph20195-bib-0137] Kitamura S . 2006. Transport of flavonoids: from cytosolic synthesis to vacuolar accumulation. In: Grotewold E , ed. The science of flavonoids. New York, NY, USA: Springer, 123–146.

[nph20195-bib-0138] Kliebenstein DJ . 2013. Making new molecules – evolution of structures for novel metabolites in plants. Current Opinion in Plant Biology 16: 112–117. 23295108 10.1016/j.pbi.2012.12.004

[nph20195-bib-0139] Kolb CA , Käser MA , Kopecký J , Zotz G , Riederer M , Pfündel EF . 2001. Effects of natural intensities of visible and ultraviolet radiation on epidermal ultraviolet screening and photosynthesis in grape leaves. Plant Physiology 127: 863–875. 11706169 PMC129258

[nph20195-bib-0140] Komatsu K , Suzuki N , Kuwamura M , Nishikawa Y , Nakatami M , Ohtawa H , Takezawa D , Seki M , Tanaka M , Taji T *et al*. 2013. Group A PP2Cs evolved in land plants as key regulators of intrinsic desiccation tolerance. Nature Communications 4: 2219. 10.1038/ncomms3219 PMC3731658 23900426

[nph20195-bib-0141] Kriechbaumer V , Botchway SW , Hawes C . 2017. Localization and interactions between Arabidopsis auxin biosynthetic enzymes in the TAA/YUC‐dependent pathway. Journal of Experimental Botany 68: 4195–4207. 10.1093/jxb/erw195 27208541

[nph20195-bib-0142] Kumar GA , Kumar S , Bhardwaj R , Swapnil P , Meena M , Seth CS , Yadav A . 2024. Recent advancements in multifaceted roles of flavonoids in plant–rhizomicrobiome interactions. Frontiers in Plant Science 14: 1297706. 38250451 10.3389/fpls.2023.1297706 PMC10796613

[nph20195-bib-0143] Kurepa J , Schull TE , Smalle JA . 2023. Friends in arms: flavonoids and the auxin/cytokinin balance in terrestrialization. Plants 23: 517. 10.3390/plants12030517 PMC9921348 36771601

[nph20195-bib-0144] Landry LC , Chapple CCS , Last R . 1995. Arabidopsis mutants lacking phenolic sunscreens exhibit enhanced ultraviolet‐b injury and oxidative damage. Plant Physiology 109: 1159–1166. 8539286 10.1104/pp.109.4.1159 PMC157646

[nph20195-bib-0145] Lauder JD , Moran EV , Hart SC . 2019. Fight or flight? Potential tradeoffs between drought defense and reproduction in conifers. Tree Physiology 39: 1071–1085. 30924877 10.1093/treephys/tpz031

[nph20195-bib-0146] Le Roy J , Huss B , Creach A , Hawkins S , Neutelings G . 2016. Glycosylation is a major regulator of phenylpropanoid availability and biological activity in plants. Frontiers in Plant Science 7: 735. 27303427 10.3389/fpls.2016.00735 PMC4880792

[nph20195-bib-0147] Lesne A . 2008. Robustness: confronting lessons from physics and biology. Biological Reviews 83: 509–532. 18823391 10.1111/j.1469-185X.2008.00052.x

[nph20195-bib-0148] Levin DA . 1971. Plant phenolics: an ecological perspective. The American Naturalist 105: 157–181.

[nph20195-bib-0149] Lewis DR , Ramirez MV , Miller ND , Vallabhaneni P , Ray WK , Helm RF , Winkel BSJ , Muday GK . 2011. Auxin and ethylene induce flavonol accumulation through distinct transcriptional networks. Plant Physiology 156: 144–164. 21427279 10.1104/pp.111.172502 PMC3091047

[nph20195-bib-0150] Li D‐D , Ni R , Wang P‐P , Shuang X , Wang P‐Y , Zhu T‐T , Sun C‐J , Liu C‐J , Lou H‐X , Cheng A‐X . 2020. Molecular basis for chemical evolution of flavones to flavonols and anthocyanins in land plants. Plant Physiology 184: 1731–1743. 33023939 10.1104/pp.20.01185 PMC7723094

[nph20195-bib-0151] Li Y , Grotewold E , Dudareva N . 2024. Enough is enough: feedback control of specialized metabolism. Trends in Plant Science 29: 514–523. 37625949 10.1016/j.tplants.2023.07.012

[nph20195-bib-0152] Lillo C , Lea US , Ruoff P . 2008. Nutrient depletion as a key factor for manipulating gene expression and product formation in different branches of the flavonoid pathway. Plant, Cell & Environment 31: 587–601. 10.1111/j.1365-3040.2007.01748.x 18031469

[nph20195-bib-0153] Lim YJ , Kwon S‐J , Eom SH . 2023. Red and blue light‐specific metabolic changes in soybean seedlings. Frontiers in Plant Science 14: 1128001. 36938020 10.3389/fpls.2023.1128001 PMC10014548

[nph20195-bib-0154] Liu S , Fang S , Cong B , Li T , Yi D , Zhang Z , Zhao L , Zhang P . 2022. The Antarctic moss *Pohlia nutans* genome provides insights into the evolution of bryophytes and the adaptation to extreme terrestrial habitats. Frontiers in Plant Science 13: 920138. 35783932 10.3389/fpls.2022.920138 PMC9247546

[nph20195-bib-0155] Lo S‐CC , Nicholson RL . 1998. Reduction of light‐induced anthocyanin accumulation in inoculated sorghum mesocotyls. Plant Physiology 116: 979–989. 9501130 10.1104/pp.116.3.979 PMC35099

[nph20195-bib-0156] Logemann E , Hahlbrock K . 2002. Crosstalk among stress responses in plants: pathogen defense overrides UV protection through an inversely regulated ACE/ACE type of light‐responsive gene promoter unit. Proceedings of the National Academy of Sciences, USA 99: 2428–2432. 10.1073/pnas.042692199 PMC122381 11842215

[nph20195-bib-0157] Lozoya E , Block A , Lois R , Hahlbrock K , Scheel D . 1991. Transcriptional repression of light‐induced flavonoid synthesis by elicitor treatment of cultured parsley cells. The Plant Journal 1: 227–234.

[nph20195-bib-0158] Lu P , Sang W‐E , Ma K‐P . 2008. Differential responses of the activities of antioxidant enzymes to thermal stresses between two invasive *Eupatorium* species in China. Journal of Integrative Plant Biology 50: 393–401. 18713373 10.1111/j.1744-7909.2007.00583.x

[nph20195-bib-0159] Maeda HA . 2019. Harnessing evolutionary diversification of primary metabolism for plant synthetic biology. Journal of Biological Chemistry 294: 16549–16566. 31558606 10.1074/jbc.REV119.006132 PMC6851331

[nph20195-bib-0160] Markham KK , Greenham K . 2021. Abiotic stress through time. New Phytologist 231: 40–46. 33780004 10.1111/nph.17367

[nph20195-bib-0161] Markham KR , Ryan KG , Bloor SJ , Mitchell KA . 1998. An increase in the luteolin: apigenin ratio in *Marchantia polymorpha* on UV‐B enhancement. Phytochemistry 48: 791–794.

[nph20195-bib-0162] Martin E , Postiglione AE , Muday GK . 2022. Reactive oxygen species function as signaling molecules in controlling plant development and hormonal responses. Current Opinion in Plant Biology 69: 102293. 36099672 10.1016/j.pbi.2022.102293 PMC10475289

[nph20195-bib-0163] Martin FM , van der Heijden MGA . 2024. The mycorrhizal symbiosis: research frontiers in genomics, ecology, and agricultural application. New Phytologist 242: 1486–1506. 38297461 10.1111/nph.19541

[nph20195-bib-0164] Martínez‐Abaigar J , Núñez‐Olivera E . 2022. Bryophyte ultraviolet‐omics: from genes to the environment. Journal of Experimental Botany 73: 4412–4426. 35274697 10.1093/jxb/erac090

[nph20195-bib-0165] Mathesius U . 2001. Flavonoids induced in cells undergoing nodule organogenesis in white clover are regulators of auxin breakdown by peroxidase. Journal of Experimental Botany 52: 419–426. 11326048 10.1093/jexbot/52.suppl_1.419

[nph20195-bib-0166] Mathesius U . 2018. Flavonoid functions in plants and their interactions with other organisms. Plants 7: 30. 29614017 10.3390/plants7020030 PMC6027123

[nph20195-bib-0167] Mazza CA , Boccalandro HE , Giordano CV , Battista D , Scopel AL , Ballaré CL . 2000. Functional significance and induction by solar radiation of ultraviolet‐absorbing sunscreens in field‐grown soybean crops. Plant Physiology 122: 117–126. 10631255 10.1104/pp.122.1.117 PMC58850

[nph20195-bib-0168] Mellor NI , Voß U , Ware A , Janes G , Barrack D , Bishopp A , Bennett MJ , Geisler M , Wells DR , Band LR . 2022. Systems approaches reveal that ABCB and PIN proteins mediate co‐dependent auxin efflux. Plant Cell 34: 2309–2327. 35302640 10.1093/plcell/koac086 PMC9134068

[nph20195-bib-0169] Michniewicz M , Zago MK , Abas L , Weijers D , Schweighofer A , Meskiene I , Heisler MG , Ohno C , Zhang J , Huang F *et al*. 2007. Antagonistic regulation of PIN phosphorylation by PP2A and PINOID directs auxin flux. Cell 130: 1044–1056. 17889649 10.1016/j.cell.2007.07.033

[nph20195-bib-0170] Milo R , Last RL . 2012. Achieving diversity in the face of constraints: lessons from metabolism. Science 336: 1663–1667. 22745419 10.1126/science.1217665

[nph20195-bib-0171] Mohana S , Ganesan M , Agilan B , Karthikeyan R , Srithar G , Beaulah MR , Ananthakrishnan D , Velmurugan D , Prasad NR , Ambudkar SV . 2016. Screening dietary flavonoids for the reversal of P‐glycoprotein‐mediated multidrug resistance in cancer. Molecular BioSystems 12: 2458–2470. 27216424 10.1039/c6mb00187d PMC4955727

[nph20195-bib-0172] Monforte L , Soriano G , Núñez‐Olivera E , Martínez‐Abaigar J . 2018. Cell compartmentation of ultraviolet‐absorbing compounds: an underexplored tool related to bryophyte ecology, phylogeny and evolution. Functional Ecology 32: 882–893.

[nph20195-bib-0173] Moody LA , Kelly S , Clayton R , Weeks Z , Emms DM , Langdale JA . 2021. *NO GAMETOPHORES 2* is a novel regulator of the 2D to 3D growth transition in the moss *Physcomitrella patens* . Current Biology 31: 555–563. 33242390 10.1016/j.cub.2020.10.077

[nph20195-bib-0174] Moore CE , Meachan‐Hensold K , Lemonnier P , Slattery RA , Benjamin C , Bernacchi CJ , Lawson T , Cavanagh AP . 2021. The effect of increasing temperature on crop photosynthesis: from enzymes to ecosystems. Journal of Experimental Botany 72: 2822–2844. 33619527 10.1093/jxb/erab090 PMC8023210

[nph20195-bib-0175] Morgan PW , Joham HE , Amin JV , Amin JW . 1966. Effect of manganese toxicity on the indoleacetic acid oxidase system of cotton. Plant Physiology 41: 718–724. 16656311 10.1104/pp.41.4.718 PMC1086411

[nph20195-bib-0176] Mravec J , Skůpa P , Bailly A , Hoyerová K , Krecek P , Bielach A , Petrásek J , Zhang J , Gaykova V , Stierhof YD *et al*. 2009. Subcellular homeostasis of phytohormone auxin is mediated by the ER‐localized PIN5 transporter. Nature 459: 1136–1140. 19506555 10.1038/nature08066

[nph20195-bib-0177] Muhlemann JK , Younts TLB , Muday GK . 2018. Flavonols control pollen tube growth and integrity by regulating ROS homeostasis during high‐temperature stress. Proceedings of the National Academy of Sciences, USA 115: E11188–E11197. 10.1073/pnas.1811492115 PMC6255205 30413622

[nph20195-bib-0178] Mutwil M . 2020. Computational approaches to unravel the pathways and evolution of specialized metabolism. Current Opinion in Plant Biology 55: 38–46. 32200228 10.1016/j.pbi.2020.01.007

[nph20195-bib-0179] Nakabayashi R , Yonekura‐Sakakibara K , Urano K , Suzuki M , Yamada Y , Nishizawa T , Matsuda F , Kojima M , Sakakibara H , Shinozaki K *et al*. 2015. Enhancement of oxidative and drought tolerance in Arabidopsis by overaccumulation of antioxidant flavonoids. The Plant Journal 77: 367–379. 10.1111/tpj.12388 PMC4282528 24274116

[nph20195-bib-0180] Neugart S , Fiol M , Schreiner M , Rohn S , Zrenner R , Jroh LW , Krunbein A . 2014. Interaction of moderate UV‐B exposure and temperature on the formation of structurally different flavonol glycosides and hydroxycinnamic acid derivatives in kale (*Brassica oleracea* var. *sabellica*). Journal of Agricultural and Food Chemistry 62: 4054–4062. 24655223 10.1021/jf4054066

[nph20195-bib-0181] Neugart S , Schreiner M . 2018. UVB and UVA as eustressors in horticultural and agricultural crops. Scientia Horticulturae 234: 370–381.

[nph20195-bib-0182] Ng JLP , Welvaert A , Wen J , Chen R , Mathesius U . 2020. The *Medicago truncatula* PIN2 auxin transporter mediates basipetal auxin transport but is not necessary for nodulation. Journal of Experimental Botany 71: 1562–1573. 31738415 10.1093/jxb/erz510

[nph20195-bib-0183] Palit S , Bhide AJ , Mohanasundaram B , Pala M , Banerjee AK . 2024. Peptides from conserved tandem direct repeats of SHORT‐LEAF regulate gametophore development in moss *P. patens* . Plant Physiology 194: 434–455. 10.1093/plphys/kiad515 37770073

[nph20195-bib-0184] Peer WA , Cheng Y , Murphy AS . 2013. Evidence of oxidative attenuation of auxin signalling. Journal of Experimental Botany 64: 2629–2639. 23709674 10.1093/jxb/ert152

[nph20195-bib-0185] Peer WA , Murphy AS . 2006. Flavonoids as signal molecules. Targets of flavonoid action. In: Grotewold E , ed. The science of flavonoids. New York, NY, USA: Springer, 239–273.

[nph20195-bib-0186] Peer WA , Murphy AS . 2007. Flavonoids and auxin transport: modulators or regulators? Trends in Plant Science 12: 556–563. 18198522 10.1016/j.tplants.2007.10.003

[nph20195-bib-0187] Peláez‐Vico MÁ , Fichman Y , Zandalinas SI , Van Breusegem F , Karpiński SM , Mittler R . 2022. ROS and redox regulation of cell‐to‐cell and systemic signaling in plants during stress. Free Radical Biology & Medicine 193: 354–362. 36279971 10.1016/j.freeradbiomed.2022.10.305

[nph20195-bib-0188] Peltzer D , Polle A . 2001. Diurnal fluctuations of antioxidative systems in leaves of field‐grown beech trees (*Fagus sylvatica*): responses to light and temperature. Physiologia Plantarum 111: 158–164.

[nph20195-bib-0189] Podolec R , Demarsy E , Ulm R . 2021. Perception and signaling of ultraviolet‐B radiation in plants. Annual Review of Plant Biology 72: 793–822. 10.1146/annurev-arplant-050718-095946 33636992

[nph20195-bib-0190] Pollastri S , Tattini M . 2011. Flavonoids: old compounds for old roles. Annals of Botany 108: 1225–1233. 21880658 10.1093/aob/mcr234 PMC3197460

[nph20195-bib-0191] Polster J , Dithmar H , Buremeister R , Friedemann G , Feucht W . 2006. Flavonoids in plant nuclei: detection by laser microdissection and pressure catapulting (LMPC), *in vivo* staining, and uv–visible spectroscopic titration. Physiologia Plantarum 128: 163–174.

[nph20195-bib-0192] Potters G , Pasternak TP , Guisez Y , Palme KJ , Jansen MAK . 2007. Stress‐induced morphogenic responses: growing out of trouble? Trends in Plant Science 12: 98–105. 17287141 10.1016/j.tplants.2007.01.004

[nph20195-bib-0193] Pratyusha DS , Sarada DVL . 2022. MYB transcription factors – master regulators of phenylpropanoid biosynthesis and diverse developmental and stress responses. Plant Cell Reports 41: 2245–2260. 36171500 10.1007/s00299-022-02927-1

[nph20195-bib-0194] Rausher MD . 2001. Co‐evolution and plant resistance to natural enemies. Nature 411: 857–864. 11459070 10.1038/35081193

[nph20195-bib-0195] Renault H , Alber A , Horst NA , Basilio Lopes A , Fich EA , Krieghauser L , Wiedemann G , Ullmann P , Herrgott L , Erhardt M *et al*. 2017. A phenol‐enriched cuticle is ancestral to lignin evolution in land plants. Nature Communications 8: 14713. 10.1038/ncomms14713 PMC5344971 28270693

[nph20195-bib-0196] Rensing SA . 2018. Great moments in evolution: the conquest of land by plants. Current Opinion in Plant Biology 42: 49–54. 29525128 10.1016/j.pbi.2018.02.006

[nph20195-bib-0197] Rensing SA . 2020. How plants conquered land. Cell 181: 964–966. 32470404 10.1016/j.cell.2020.05.011

[nph20195-bib-0198] Rice‐Evans CA , Miller NJ , Papanga G . 1996. Structure‐antioxidant activity relationships of flavonoids and phenolic acids. Free Radical Biology & Medicine 20: 933–956. 8743980 10.1016/0891-5849(95)02227-9

[nph20195-bib-0199] Richter AS , Nägele T , Grimm B , Kaufmann K , Schroda M , Leister D , Kleine T . 2023. Retrograde signaling in plants: a critical review focusing on the GUN pathway and beyond. Plant Communications 9: 1005101. 10.1016/j.xplc.2022.100511 PMC9860301 36575799

[nph20195-bib-0200] Richter AS , Tohge T , Fernie AR , Grimm B . 2020. The genomes uncoupled dependent signalling pathway coordinates plastid biogenesis with the synthesis of anthocyanins. Philosophical Transactions of the Royal Society of London. Series B: Biological Sciences 375: 20190403. 32362259 10.1098/rstb.2019.0403 PMC7209955

[nph20195-bib-0201] Rieseberg TM , Dadras A , Fürst‐Jansen JMR , Ashok AD , Darienko T , de Vries S , Irisarri I , de Vries J . 2021. Crossroads in the evolution of plant specialized metabolism. Seminars in Cell & Developmental Biology 134: 37–58. 10.1016/j.semcdb.2022.03.004 35292191

[nph20195-bib-0202] Robberecht R , Caldwell MM . 1978. Leaf epidermal transmittance of ultraviolet radiation and its implications for plant sensitivity to ultraviolet‐radiation induced injury. Oecologia 32: 277–287. 28309272 10.1007/BF00345107

[nph20195-bib-0203] Robson TM , Klem K , Urban O , Jansen MAK . 2015. Re‐interpreting plant morphological responses to UV‐B radiation. Plant, Cell & Environment 38: 856–866. 10.1111/pce.12374 24890713

[nph20195-bib-0204] Roepke J , Bozzo GG . 2015. *Arabidopsis thaliana* β‐glucosidase BGLU15 attacks flavonol 3‐*O*‐β‐glucoside‐7‐*O*‐α‐rhamnosides. Phytochemistry 109: 14–24. 25468534 10.1016/j.phytochem.2014.10.028

[nph20195-bib-0205] Rousseaux MC , Julkunen‐Tiitto R , Searles PS , Scopel AL , Ballaré CL . 2004. Solar UV‐B radiation affects leaf quality and insect herbivory in the southern beech tree *Nothofagus Antarctica* . Oecologia 138: 505–512. 14740287 10.1007/s00442-003-1471-5

[nph20195-bib-0206] Rozema J , Björn OL , Bornman JF , Gaberscik A , Häder D‐P , Trost T , Germ M , Klisch M , Gröniger A , Sinha RP *et al*. 2002. The role of UV‐B radiation in aquatic and terrestrial ecosystems – an experimental and functional analysis of the evolution of UV‐absorbing compounds. Journal of Photochemistry and Photobiology B 66: 2–12. 10.1016/s1011-1344(01)00269-x 11849977

[nph20195-bib-0207] Rozema J , van de Staaij J , Björn LO , Caldwell M . 1997. UV‐B as an environmental factor in plant life: stress and regulation. Trends in Ecology & Evolution 12: 22–28. 21237957 10.1016/s0169-5347(96)10062-8

[nph20195-bib-0208] Ryan KG , Swinny EE , Winefield C , Markham KE . 2001. Flavonoids and UV photoprotection in Arabidopsis mutants. Zeitschrift für Naturforschung B 56c: 745–754. 10.1515/znc-2001-9-1013 11724379

[nph20195-bib-0209] Sachdev S , Ansari SA , Ansari MI , Fujita M , Hasanuzzaman M . 2021. Abiotic stress and reactive oxygen species: generation, signaling, and defense mechanisms. Antioxidants 10: 277. 33670123 10.3390/antiox10020277 PMC7916865

[nph20195-bib-0210] Salminen J‐P , Karonen M . 2011. Chemical ecology of tannins and other phenolics: we need a change in approach. Functional Ecology 25: 325–338.

[nph20195-bib-0211] Saracini E , Tattini M , Traversi ML , Vincieri FF , Pinelli P . 2005. Simultaneous LC‐DAD and LC‐MS determination of ellagitannins, flavonoid glycosides, and acyl‐glycosyl flavonoids in *Cistus salvifolius* L. leaves. Chromatographia 62: 245–249.

[nph20195-bib-0212] Schneider GF , Coley PD , Younkin GC , Forrister DL , Mills AG , Kursar TA . 2019. Phenolics lie at the centre of functional versatility in the responses of two phytochemically diverse tropical trees to canopy thinning. Journal of Experimental Botany 20: 5853–5864. 10.1093/jxb/erz308 PMC6812699 31257446

[nph20195-bib-0213] Schnitzler J‐P , Jungblut TP , Heller W , Kofferlein M , Hutzler P , Heinzmann U , Schmelzer E , Ernst D , Langebertels C , Sandermann H Jr . 1996. Tissue localization of u.v.‐B‐screening pigments and of chalcone synthase mRNA in needles of Scots pine seedlings. New Phytologist 132: 247–258.

[nph20195-bib-0214] Schreiber M , Rensing SA , Gould SB . 2022. The greening ashore. Trends in Plant Science 27: 847–857. 35739050 10.1016/j.tplants.2022.05.005

[nph20195-bib-0215] Segarra‐Medina C , Alseekh S , Fernie AR , Rambla JL , Pérez‐Clemente RM , Gómez‐Cádenas A , Zandalinas SI . 2023. Abscisic acid promotes plant acclimation to the combination of salinity and high light stress. Plant Physiology and Biochemistry 203: 108008. 37690143 10.1016/j.plaphy.2023.108008

[nph20195-bib-0216] Serrano M , Kanehara K , Torres M , Yamada K , Tintor N , Kombrink E , Schulze‐Lefeer P , Saijo Y . 2012. Repression of sucrose/ultraviolet B light‐induced flavonoid accumulation in microbe‐associated molecular pattern‐triggered immunity in Arabidopsis. Plant Physiology 158: 408–422. 22080602 10.1104/pp.111.183459 PMC3252079

[nph20195-bib-0217] Shitan N , Yazaki K . 2020. Dynamism of vacuoles toward survival strategy in plants. Biochimica et Biophysica Acta – Biomembranes 1862: 183127. 31738903 10.1016/j.bbamem.2019.183127

[nph20195-bib-0218] Siipola SM , Kotilanen T , Sipari N , Morales LO , Lindfors AV , Robson TM , Aphalo PG . 2016. Epidermal UV‐A absorbance and whole‐leaf flavonoid composition in pea respond more to solar blue light than to solar UV radiation. Plant, Cell & Environment 38: 941–952. 10.1111/pce.12403 25040832

[nph20195-bib-0219] Singh G , Agrawal H , Bednarek P . 2023. Specialized metabolites as versatile tools in shaping plant–microbe associations. Molecular Plant 16: 122–144. 36503863 10.1016/j.molp.2022.12.006

[nph20195-bib-0220] Singh P , Arif Y , Bajguz A , Hayat S . 2021. The role of quercetin in plants. Plant Physiology and Biochemistry 166: 10–19. 34087741 10.1016/j.plaphy.2021.05.023

[nph20195-bib-0221] Skokan R , Medvecka E , Viane T , Vosolsobê S , Zwiewka M , Muller K , Skupa P , Karadi M , Zhang Y , Janacek DP *et al*. 2019. PIN‐driven auxin transport emerged early in streptophyte evolution. Nature Communications 5: 1114–1119. 10.1038/s41477-019-0542-5 31712756

[nph20195-bib-0222] Soengas P , Rodríguez VM , Velasco P , Cartea ME . 2018. Effect of temperature stress on antioxidant defenses in *Brassica oleracea* . ACS Omega 3: 5237–5243. 30023910 10.1021/acsomega.8b00242 PMC6044755

[nph20195-bib-0223] Song Q , Kong L , Yang J , Wang X , Zhao Z , Zhang Y , Xu C , Fan C , Luo K . 2024. The IAA17.1/HSFA5a module enhances salt tolerance in *Populus tomentosa* by regulating flavonol biosynthesis and ROS levels in lateral roots. New Phytologist 241: 592–606. 37974487 10.1111/nph.19382

[nph20195-bib-0224] Song Z , Lai X , Chen H , Wang L , Pang X , Hao Y , Lu W , Chen W , Zhu X , Li X . 2022. Role of MaABI5‐like in abscisic acid‐induced cold tolerance of ‘Fenjiao’ banana fruit. Horticulture Research 9: uhac130. 36936195 10.1093/hr/uhac130 PMC10021067

[nph20195-bib-0225] Soriano G , Clix C , Heilmann M , Núñez‐Olivera E , Martínez‐Abaigar J , Jenkins GI . 2018. Evolutionary conservation of structure and function of the UVR8 photoreceptor from the liverwort *Marchantia polymorpha* and the moss *Physcomitrella patens* . New Phytologist 217: 151–162. 28892172 10.1111/nph.14767

[nph20195-bib-0226] Stafford HA . 1965. Flavonoids and related phenolic compounds produced in the first internode of *Sorghum vulgare* Pers. in darkness and in light. Plant Physiology 40: 130–138. 5831095 10.1104/pp.40.1.130 PMC550252

[nph20195-bib-0227] Stafford HA . 1991. Flavonoid evolution: an enzymic approach. Plant Physiology 96: 680–685. 16668242 10.1104/pp.96.3.680 PMC1080830

[nph20195-bib-0228] Steemans P , Le Hérissé A , Melvin J , Miller MA , Paris F , Verniers J , Wellman CH . 2009. Origin and radiation of the earliest vascular land plants. Science 324: 353. 19372423 10.1126/science.1169659

[nph20195-bib-0229] Steppuhun A , Baldwin IT . 2008. Induced defenses and the cost‐benefit paradigm. In: Schaller A , ed. Induced plant resistance to herbivory. New York, NY, USA: Springer Science, 61–87.

[nph20195-bib-0230] Sugimoto K , Zager JJ , St Aubin B , Lange BM , Howe GA . 2022. Flavonoid deficiency disrupts redox homeostasis and terpenoid biosynthesis in glandular trichomes of tomato. Plant Physiology 188: 1450–1468. 34668550 10.1093/plphys/kiab488 PMC8896623

[nph20195-bib-0231] Swain T . 1975. Evolution of flavonoid compounds. In: Harborne JB , Mabry TJ , Mabry H , eds. The flavonoids. Boston, MA, USA: Springer, 1096–1129.

[nph20195-bib-0232] Swain T . 1977. Secondary compounds as protective agents. Annual Review of Plant Physiology 28: 479–501.

[nph20195-bib-0233] Tang H , Lu K‐J , Zhang Y , Cheng Y‐L , Tu S‐L , Friml J . 2024. Divergence of trafficking and polarization mechanisms for PIN auxin transporters during land plant evolution. Plant Communications 5: 100669. 37528584 10.1016/j.xplc.2023.100669 PMC10811345

[nph20195-bib-0234] Tattini M , Galardi C , Pinelli P , Massai R , Remorini D , Agati G . 2004. Differential accumulation of flavonoids and hydroxycinnamates in leaves of *Ligustrum vulgare* under excess light and drought stress. New Phytologist 163: 547–561. 33873733 10.1111/j.1469-8137.2004.01126.x

[nph20195-bib-0235] Tattini M , Gravano E , Pinelli P , Mulinacci N , Romani A . 2000. Flavonoids accumulate in leaves and glandular trichomes of *Phillyrea latifolia* exposed to excess solar radiation. New Phytologist 148: 69–77. 33863030 10.1046/j.1469-8137.2000.00743.x

[nph20195-bib-0236] Tattini M , Loreto F , Fini A , Guidi L , Brunetti C , Velikova V , Gori A , Ferrini F . 2015. Isoprenoids and phenylpropanoids are part of the antioxidant defense orchestrated daily by drought‐stressed *Platanus* × *acerifolia* plants during Mediterranean summers. New Phytologist 207: 613–626. 25784134 10.1111/nph.13380

[nph20195-bib-0237] Tattini M , Matteini P , Saracini E , Traversi ML , Giordano C , Agati G . 2007. Morphology and biochemistry of non‐glandular trichomes in *Cistus salvifolius* L. leaves growing in extreme habitats of the Mediterranean basin. Plant Biology 9: 411–419. 17143807 10.1055/s-2006-924662

[nph20195-bib-0238] Taulavuori K , Pyysalo A , Taulavuori E , Julkunen‐Tiitto R . 2018. Responses of phenolic acid and flavonoid synthesis to blue and blue‐violet light depends on plant species. Environmental and Experimental Botany 150: 183–187.

[nph20195-bib-0239] Taylor LP , Grotewold E . 2005. Flavonoids as developmental regulators. Current Opinion in Plant Biology 8: 317–323. 15860429 10.1016/j.pbi.2005.03.005

[nph20195-bib-0240] Teale WD , Pasternal T , Dal Bosco C , Dovzhenko A , Kratzat K , Bildl E , Schwörer M , Falk T , Ruperti B , Schaefer JV . 2020. Flavonol‐mediated stabilization of PIN efflux complexes regulates polar auxin transport. EMBO Journal 40: e104416. 33185277 10.15252/embj.2020104416 PMC7780147

[nph20195-bib-0241] Tian B , Pei Y , Huang W , Ding J , Siemann E . 2021. Increasing flavonoid concentrations in root exudates enhance associations between arbuscular mycorrhizal fungi and an invasive plant. The ISME Journal 15: 1919–1930. 33568790 10.1038/s41396-021-00894-1 PMC8245413

[nph20195-bib-0242] Tohge T , Perez de Souza L , Fernie AR . 2018. On the natural diversity of phenylacylated‐flavonoid and their *in planta* function under conditions of stress. Phytochemistry Reviews 17: 279–290. 29755304 10.1007/s11101-017-9531-3 PMC5932100

[nph20195-bib-0243] Tohge T , Wendenburg R , Ishihara H , Nakabayashi R , Watanabe M , Sulpice R , Hoefgen R , Takayama H , Saito K , Stitt M *et al*. 2016. Characterization of a recently evolved flavonol‐phenylacyltransferase gene provides signatures of natural light selection in *Brassicaceae* . Nature Communications 7: 12399. 10.1038/ncomms12399 PMC4996938 27545969

[nph20195-bib-0244] Tripp EA , Zhuang Y , Schreiber M , Stone H , Berardi EA . 2018. Evolutionary and ecological drivers of plant flavonoids across a large latitudinal gradient. Molecular Phylogenetics and Evolution 128: 147–161. 30017824 10.1016/j.ympev.2018.07.004

[nph20195-bib-0245] Uehara A , Shimoda K , Murai Y , Ivashina T . 2018. Flavonoid aglycones and glycosides from the leaves of some Japanese *Artemisia* species. Natural Products Communications 13: 551–554.

[nph20195-bib-0246] Ung KL , Winkler M , Schulz L , Kolb M , Janacek DP , Dedic E , Stokes DL , Hammes UZ , Pedersen BP . 2022. Structures and mechanism of the plant PIN‐FORMED auxin transporter. Nature 609: 605–610. 35768502 10.1038/s41586-022-04883-y PMC9477730

[nph20195-bib-0247] Venegas‐Molina J , Molina‐Hidalgo FJ , Clicque E , Goosens A . 2021. Why and how to dig into plant metabolite‐protein interactions. Trends in Plant Science 26: 472–483. 33478816 10.1016/j.tplants.2020.12.008

[nph20195-bib-0248] Viaene T , Landberg K , Thelander M , Medvecka E , Pederson E , Feraru E , Cooper ED , Karimi M , Delwiche CF , Ljung K *et al*. 2014. Directional auxin transport mechanisms in early diverging land plants. Current Biology 24: 2786–2791. 25448004 10.1016/j.cub.2014.09.056

[nph20195-bib-0249] de Vries J , de Vries S , Slamovits CH , Rose LA , Archibald JM . 2017. How embryophytic is the biosynthesis of phenylpropanoids and their derivatives in Streptophyte algae? Plant & Cell Physiology 58: 934–945. 28340089 10.1093/pcp/pcx037

[nph20195-bib-0250] de Vries S , Furst‐Jansen JMR , Irisarri I , Ashok AD , Ischebeck T , Feussner K , Abreu IN , Petersen M , Feussner I , de Vries J . 2021. The evolution of the phenylpropanoid pathway entailed pronounced radiations and divergences of enzyme families. The Plant Journal 107: 975–1002. 34165823 10.1111/tpj.15387

[nph20195-bib-0251] Wagner A . 2011. The molecular origins of evolutionary innovations. Trends in Genetics 27: 397–410. 21872964 10.1016/j.tig.2011.06.002

[nph20195-bib-0252] Wang C , Liu Y , Li S‐S , Han G‐Z . 2015. Insights into the origin and evolution of the plant hormone signaling machinery. Plant Physiology 167: 872–886. 25560880 10.1104/pp.114.247403 PMC4348752

[nph20195-bib-0253] Wang N , Liu W , Yu L , Guo Z , Chen Z , Jiang S , Xu H , Fang H , Wang Y , Zhang Z *et al*. 2020. HEAT SHOCK FACTOR A8a modulates flavonoid synthesis and drought tolerance. Plant Physiology 184: 1273–1290. 32958560 10.1104/pp.20.01106 PMC7608180

[nph20195-bib-0254] Wang S , Alseekh S , Fernie AR , Luo J . 2019. The structure and function of major plant metabolite modifications. Molecular Plant 12: 899–919. 31200079 10.1016/j.molp.2019.06.001

[nph20195-bib-0255] Watkins JM , Chapman JM , Muday GK . 2017. Abscisic acid‐induced reactive oxygen species are modulated by flavonols to control stomata aperture. Plant Physiology 175: 1807–1825. 29051198 10.1104/pp.17.01010 PMC5717730

[nph20195-bib-0256] Watkins JM , Hechler PJ , Muday GK . 2014. Ethylene‐induced flavonol accumulation in guard cells suppresses reactive oxygen species and moderates stomatal aperture. Plant Physiology 164: 1707–1717. 24596331 10.1104/pp.113.233528 PMC3982735

[nph20195-bib-0257] Wellmann E . 1976. Specific ultraviolet effects in plant morphogenesis. Photochemistry and Photobiology 24: 659–660. 798216 10.1111/j.1751-1097.1976.tb06889.x

[nph20195-bib-0258] Wen W , Alseekh S , Fernie AR . 2020. Conservation and diversification of flavonoid metabolism in the plant kingdom. Current Opinion in Plant Biology 55: 100–108. 32422532 10.1016/j.pbi.2020.04.004

[nph20195-bib-0259] Weng J‐K , Lynch JH , Matos JO , Dudareva N . 2021. Adaptive mechanisms of plant specialized metabolism connecting chemistry to function. Nature Chemical Biology 17: 1037–1045. 34552220 10.1038/s41589-021-00822-6

[nph20195-bib-0260] Williams RJ , Spencer JPE , Rice‐Evans CA . 2004. Flavonoids: antioxidants or signalling molecules? Free Radical Biology & Medicine 36: 838–849. 15019969 10.1016/j.freeradbiomed.2004.01.001

[nph20195-bib-0261] Williamson G . 2002. The use of flavonoid aglycones in *in vitro* systems to test biological activities: based on bioavailability data, is this a valid approach? Phytochemistry Reviews 1: 215–222.

[nph20195-bib-0262] Wink M . 1999. Functions of plant secondary metabolites and their exploitation in biotechnology. Annual plant reviews, vol. 3. Sheffield, UK: Sheffield Academic Press.

[nph20195-bib-0263] Wolf L , Rizzini L , Stracke R , Ulm R , Rensing SA . 2010. The molecular and physiological responses of *Physcomitrella patens* to ultraviolet‐B radiation. Plant Physiology 153: 1123–1134. 20427465 10.1104/pp.110.154658 PMC2899899

[nph20195-bib-0264] Wollenweber E , Dörr M , Christ M . 2011. Flavonoid aglycones from the leaf and stem exudates of some Geraniaceae species. Natural Products Communications 6: 15–16. 21366037

[nph20195-bib-0265] Xu B , Taylor L , Pucker B , Feng T , Glover BJ , Brockington SF . 2021. The land plant‐specific MIXTA‐MYB lineage is implicated in the early evolution of the plant cuticle and the colonization of land. New Phytologist 229: 2324–2338. 33051877 10.1111/nph.16997

[nph20195-bib-0266] Xu W , Dubos C , Lepiniec L . 2015. Transcriptional control of flavonoid biosynthesis by MYB–bHLH–WDR complexes. Trends in Plant Science 20: 176–185. 25577424 10.1016/j.tplants.2014.12.001

[nph20195-bib-0267] Yamasaki H , Sakihama Y , Ikebara N . 1997. Flavonoid‐peroxidase reaction as a detoxification mechanism of plant cells against H_2_O_2_ . Plant Physiology 115: 1405–1412. 12223873 10.1104/pp.115.4.1405 PMC158605

[nph20195-bib-0268] Yonekura‐Sakakibara K , Higashi Y , Nakabayashi R . 2019. The origin and evolution of plant flavonoid metabolism. Frontiers in Plant Science 10: 943. 31428108 10.3389/fpls.2019.00943 PMC6688129

[nph20195-bib-0269] Zhang D , Jian C , Huang C , Wen D , Lu J , Chen S , Zhang T , Shi Y , Xue J , Ma W *et al*. 2018. The light‐induced transcription factor FtMYB116 promotes accumulation of rutin in *Fagopyrum tataricum* . Plant, Cell & Environment 42: 1340–1351. 10.1111/pce.13470 30375656

[nph20195-bib-0270] Zhang J , Lin JL , Harris C , Peer WA . 2016. DAO1 catalyzes temporal and tissue‐specific oxidative inactivation of auxin in *Arabidopsis thaliana* . Proceedings of the National Academy of Sciences, USA 113: 11010–11015. 10.1073/pnas.1604769113 PMC5047167 27651492

[nph20195-bib-0271] Zhang J , Peer WA . 2017. Auxin homeostasis: the DAO of catabolism. Journal of Experimental Botany 68: 3145–3154. 28666349 10.1093/jxb/erx221

[nph20195-bib-0272] Zhang J , Subramanian S , Stacey G , Yu O . 2009. Flavones and flavonols play distinct critical roles during nodulation of *Medicago truncatula* by *Sinorhizobium meliloti* . The Plant Journal 57: 171–183. 18786000 10.1111/j.1365-313X.2008.03676.x

